# Multi-omic analysis identifies metabolic biomarkers for the early detection of breast cancer and therapeutic response prediction

**DOI:** 10.1016/j.isci.2024.110682

**Published:** 2024-08-05

**Authors:** Huajie Song, Xiaowei Tang, Miao Liu, Guangxi Wang, Yuyao Yuan, Ruifang Pang, Chenyi Wang, Juntuo Zhou, Yang Yang, Mengmeng Zhang, Yan Jin, Kewei Jiang, Shu Wang, Yuxin Yin

**Affiliations:** 1 Department of Pathology, Institute of Systems Biomedicine, School of Basic Medical Sciences, Peking-Tsinghua Center for Life Sciences, Peking University Health Science Center, Beijing 100191, China; 2 Breast Center, Peking University People’s Hospital, Beijing 100044, China; 3 Institute of Precision Medicine, Peking University Shenzhen Hospital, Shenzhen 518036, P.R. China; 4 Department of Gastroenterological Surgery, Peking University People’s Hospital, Beijing 100044, China

**Keywords:** Cancer, Metabolomics, Machine learning

## Abstract

Reliable blood-based tests for identifying early-stage breast cancer remain elusive. Employing single-cell transcriptomic sequencing analysis, we illustrate a close correlation between nucleotide metabolism in the breast cancer and activation of regulatory T cells (Tregs) in the tumor microenvironment, which shows distinctions between subtypes of patients with triple-negative breast cancer (TNBC) and non-TNBC, and is likely to impact cancer prognosis through the A2AR-Treg pathway. Combining machine learning with absolute quantitative metabolomics, we have established an effective approach to the early detection of breast cancer, utilizing a four-metabolite panel including inosine and uridine. This metabolomics study, involving 1111 participants, demonstrates high accuracy across the training, test, and independent validation cohorts. Inosine and uridine prove predictive of the response to neoadjuvant chemotherapy (NAC) in patients with TNBC. This study deepens our understanding of nucleotide metabolism in breast cancer development and introduces a promising non-invasive method for early breast cancer detection and predicting NAC response in patients with TNBC.

## Highlights

• Nucleotide metabolism affects Tregs in breast cancer microenvironment
• Four-metabolite panel detects early breast cancer accurately
• Inosine and uridine predict TNBC chemotherapy response
• Machine learning refines plasma metabolomics for breast cancer diagnosis

## Graphical abstract

**Figure:**
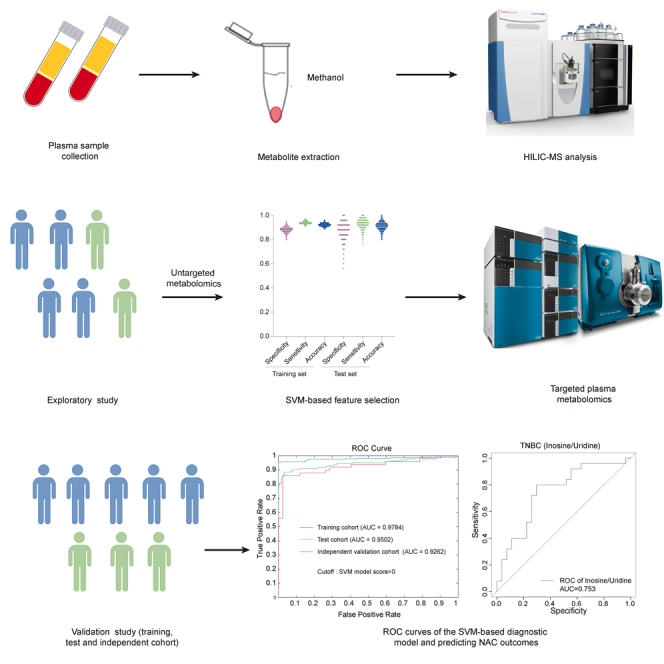

## Introduction

Breast cancer (BC) is the most commonly diagnosed cancer and the leading cause of cancer death in women globally.^1^ According to the latest global cancer burden data released by the World Health Organization’s International Agency for Research on Cancer (IARC), there are approximately 2.30 million new BC cases and 670,000 BC-related deaths worldwide in 2022.^1^ Breast cancer could be classified into multiple molecular subtypes according to various criteria, based on expression of estrogen receptor (ER), progesterone receptor (PR) and human epidermal growth factor receptor 2 (HER2) or transcription of PAM50 gene signatures.^2^^,^^3^ Consequently, the substantial heterogeneity of BC makes it complicated to choose proper therapeutic strategies in clinical settings.^4^

As the conventional screening approach of BC, mammography has been controversial in terms of cost-effectiveness, false-positive rate, and overdiagnosis.^5^ Though screening by mammography markedly promotes survival in high-income countries, individuals from low-income countries obtain far fewer benefits due to limited access to screening programs, demonstrating an urgent need for improved screening approaches.^6^^,^^7^ Recent evidence has shown the possibility of detecting DNA methylation, proteins, and metabolites in blood for the early detection of BC.^8^^,^^9^^,^^10^^,^^11^ However, the robustness of these methods still needs to be validated by targeted methods in larger cohorts.

Recent advancements in anticancer treatments have expanded treatment options for patients with breast cancer, offering targeted, personalized, and less toxic therapies. Immunotherapy, targeted therapy, and new treatment combinations have emerged and provided important references for precise treatment.^12^^,^^13^^,^^14^^,^^15^^,^^16^ Neoadjuvant chemotherapy (NAC) has become the treatment of choice for patients with inflammatory breast cancer, especially for patients with HER2-positive or TNBC.^17^ Given the complexity of the therapeutic strategies of BC and the corresponding impact on prognosis, recent studies have also been dedicated to developing predictive markers for the treatment response of various therapies, in which genomic, proteomic, and immune-related signatures have been identified.^18^^,^^19^^,^^20^^,^^21^ However, studies focusing on predicting the response to NAC in patients with BC using plasma metabolic signatures are relatively few.^18^^,^^22^

The emergence of single-cell sequencing analysis has made it possible to systematically understand the cellular landscape of tumor microenvironment in BC of various molecular subtypes. Additionally, metabolomics has also emerged as a valuable tool for comprehensive analysis of global metabolic changes in living organisms.^23^^,^^24^ Through analyzing small molecules that change in response to biological perturbations, metabolomics can provide important insights into disease states and offer more opportunities for biomarker identification.^25^^,^^26^^,^^27^ In this study, we sought to integrate single-cell RNA sequencing (scRNA-seq) and metabolomic methods for a better understanding of the pathogenesis of BC and identified the dysregulation of nucleotide metabolism of various degrees in different subtypes of BC. Machine learning (ML), an artificial intelligence (AI) technique widely utilized in multiple aspects of medical research,^28^^,^^29^^,^^30^ was also employed to identify the most relevant subset of metabolic biomarkers, establishing a highly accurate model for the early detection of BC. Our further investigation also revealed a potent role of plasma nucleoside markers, inosine and uridine, in predicting outcomes of neoadjuvant NAC, providing potential guidance for clinical decisions.

## Results

### Overview of the study scheme

**Figure 1:**
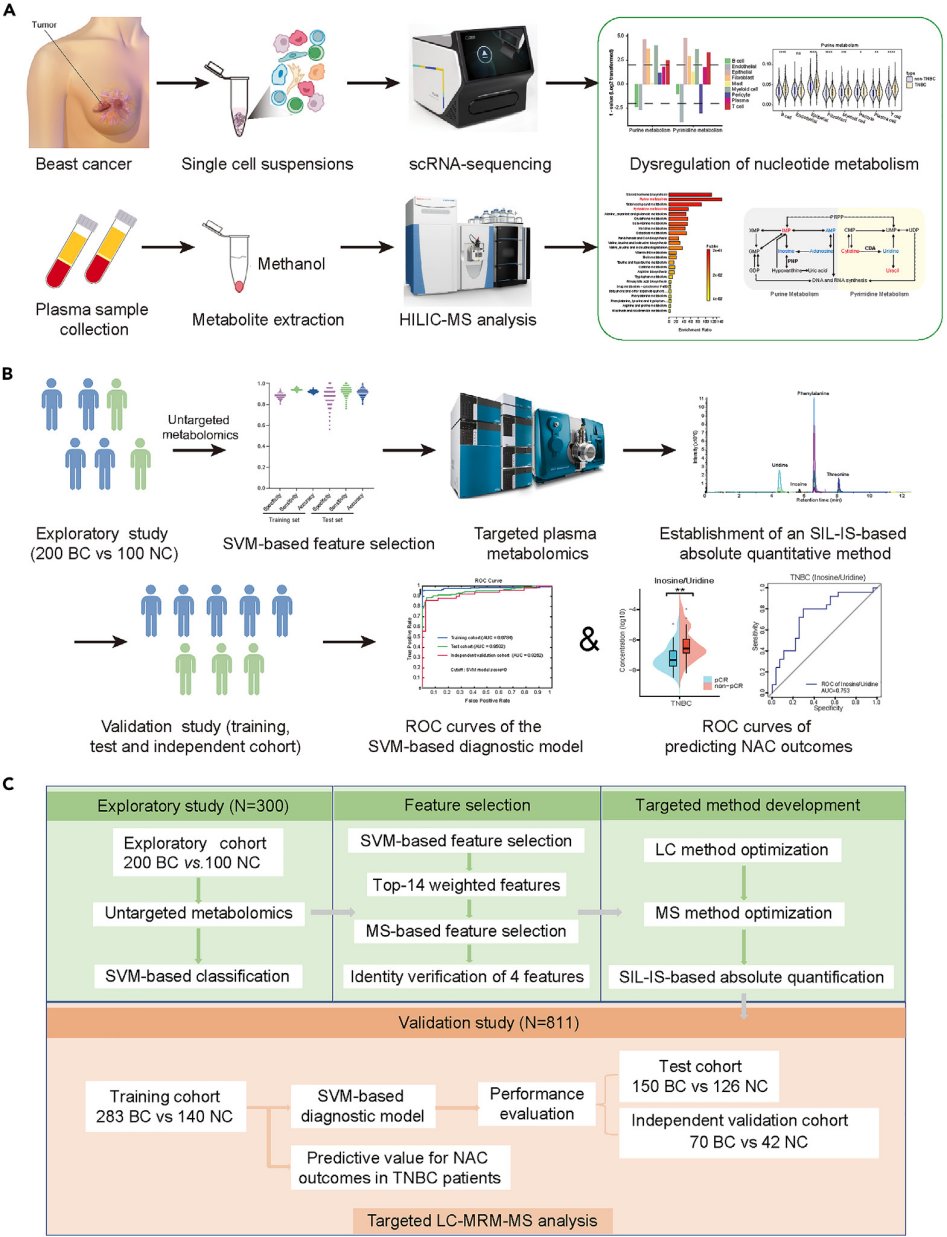
Study workflow overview (A) Schematic representation of scRNA-seq analyses and plasma untargeted metabolomics. (B) Study strategy of SVM-based diagnostic model and prediction of NAC outcomes. (C) Experimental strategy of the exploratory study and the validation study for metabolomics analysis.

The workflow and research strategy for this study were developed and depicted in Figures 1A–1C. To obtain a comprehensive understanding of the global metabolic changes of BC, both in the tumor microenvironment and circulation, scRNA-seq analysis of cancer tissue and metabolomic analysis of plasma samples were performed. Metabolic pathways of substantial enrichment were analyzed (Figure 1A). Given recent reports on new regulatory roles of nucleosides and nucleotides in anticancer immunity,^31^^,^^32^ critical pathways including pyrimidine and purine metabolism attracted our attention. To achieve our objective of screening for plasma metabolite markers of early-stage BC, AI-based methods were thereby employed to select important targets and targeted quantitation methods were established to develop predictive models (Figure 1B).

For plasma metabolic profiling, we initially conducted untargeted metabolomics analysis in an exploratory cohort comprising 200 BC and 100 normal controls (NC) using liquid chromatography-mass spectrometry (LC-MS) in data-dependent acquisition (DDA) mode (Figures 1C and S1A). Subsequently, a feature selection procedure based on a support vector machine (SVM) was used to pick out truly important features distinguishing BC from NC, and finally, 4 metabolites were selected as a panel of diagnostic biomarkers. Following this, an absolute quantification method utilizing stable isotope labeled-internal standards (SIL-IS) was developed to simultaneously quantify these four metabolites. This targeted approach was applied to a training cohort (*n* = 423, 283 BC and 140 NC), a test cohort (*n* = 276, 150 BC and 126 NC), and an independent validation cohort (*n* = 112, 70 BC and 42 NC) (Figures 1C and S1A). Lastly, an independent cohort of patients subjected to NAC was established for the evaluation of predictive performance on therapeutic outcomes.

### Single-cell transcriptome analysis reveals the impact of nucleotide metabolism on breast cancer development

**Figure 2:**
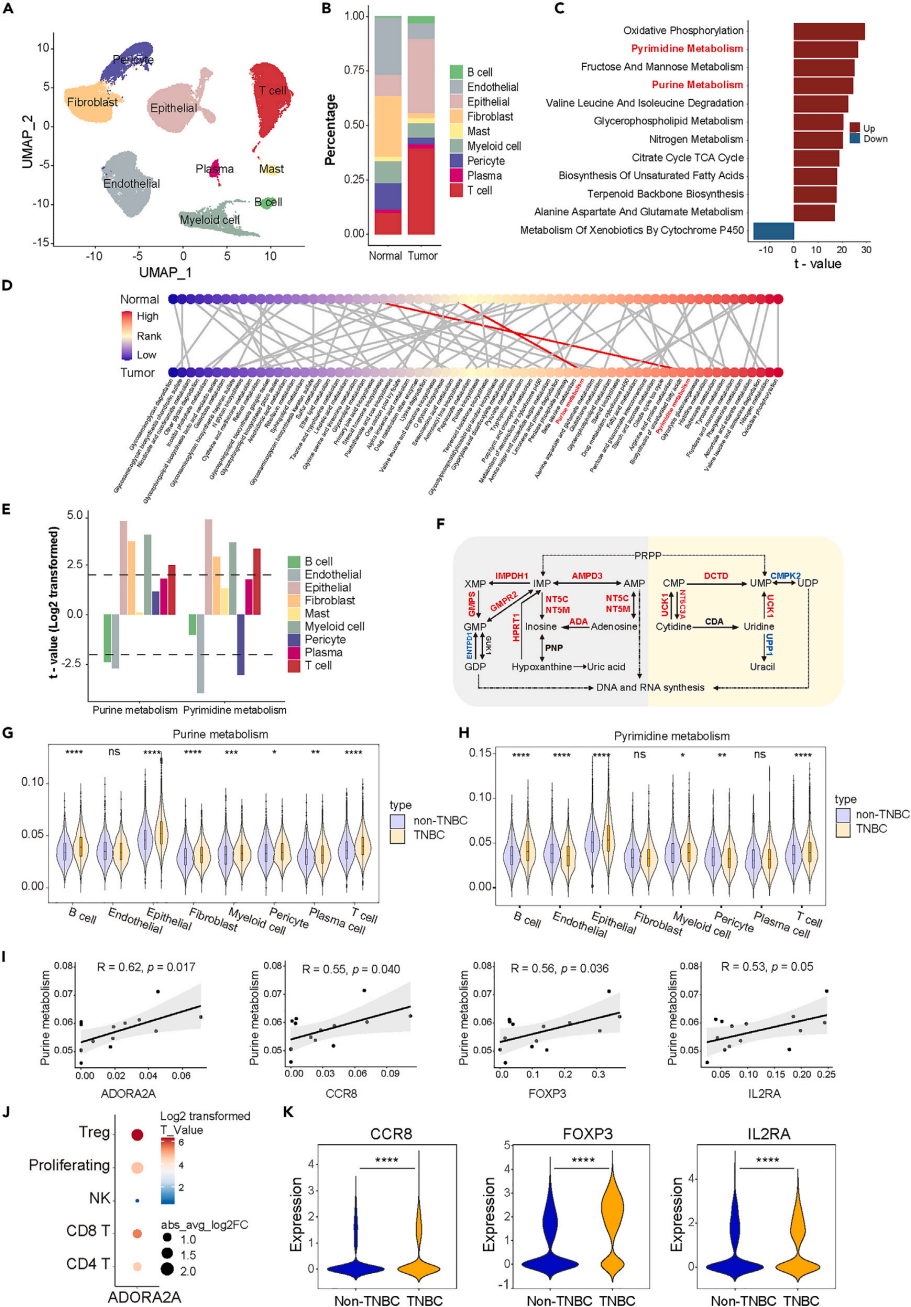
scRNA-seq analyses of BC tissues (A) Cell populations identified in human breast tissues. The UMAP visualization of 51,549 single cells from normal tissue (*n* = 4) and BC tissue (*n* = 5) samples, with nine major cell clusters identified and labeled. (B) Average proportion of different cell types in normal breast samples (*n* = 4) and BC tissues (*n* = 5). (C) AUCell analyses of up-regulated and down-regulated pathways. The top 12 enriched KEGG terms in metabolism-related gene sets are shown (absolute t > 16). (D) Expression changes of metabolic pathways in tumor and normal epithelial cells. Metabolic pathways are ordered by the log-fold change in normal epithelial cells (upper panel) and tumor cells (lower panel), respectively. The same pathway in the normal and BC tissues is connected. Each node represents a single pathway and the node color represents its relative expression levels in normal epithelial cells or tumor cells compared to other cell types. (E) Comparison of purine metabolism and pyrimidine metabolism in each cell type between BC and normal breast tissues. Each bar color represents a different cell type, and the bars above the dotted line indicate significant differences (absolute t > 2). (F) Dysregulated metabolic genes involved in purine metabolism (gray) and pyrimidine metabolism (yellow) in tumor epithelial cells. Up-regulated genes in red, down-regulated genes in blue. (G and H) Comparison of purine metabolism (G) and pyrimidine metabolism (H) between TNBC and non-TNBC samples in each cell type (*t*-test, ∗*p* < 0.05, ∗∗*p* < 0.01, ∗∗∗*p* < 0.001, ∗∗∗∗*p* < 0.0001). (I) The correlation between purine metabolism in tumor epithelial cells and the expression of nucleoside receptor A2AR on T cells, as well as three Treg cell markers. (J) The dot plot illustrates the expression change of A2AR between TNBC and non-TNBC samples in various T cell types. (K) The dot plot illustrates the expression change of three Treg cell markers in Treg cells between TNBC and non-TNBC samples (*t*-test, ∗∗∗∗*p* < 0.0001).

For a comprehensive understanding of metabolic portraits in BC development, we performed droplet-based scRNA-seq analysis on freshly excised primary tumor specimens and adjacent normal breast tissues. After quality control filtering, a total of 51,549 cells were retained from nine samples sourced from five patients (Table S1). Among these cells, 26,410 cells (51.2%) originated from five BC tissues and 25,139 (48.8%) from four normal breast tissues. Batch correction methods were applied to the transcriptomic datasets, and we identified nine primary cell lineages distinguished by well-established markers, encompassing T cells, B cells, plasma cells, myeloid cells, mast cells, fibroblasts, endothelial cells, pericytes, and epithelial cells (Figures 2A and S2A). Additionally, we observed augmented relative proportions of T lymphocytes, B cells, and epithelial cells in BC tissues, while diminished relative proportions of endothelial cells, fibroblasts, and pericytes compared to normal breast tissues, presenting an activated immune status in BC tissues (Figure 2B). To distinguish tumor cells from normal epithelial cells, the main copy number variation (CNV) events in each cell were determined from its transcriptomic profile.^33^ As depicted in Figure S2B, epithelial cells from tumor tissues exhibited large-scale CNVs when compared to their normal epithelial counterparts. Furthermore, cells from the same patient shared similar CNV status, suggesting a high degree of intertumor heterogeneity.

Seventy metabolism pathways (see STAR Methods section) were compared in each of the nine cell lineages between BC and normal breast tissues. A focused analysis of tumor cells and normal epithelial cells revealed substantial alteration in metabolism pathways, with the top 12 perturbed metabolic pathways highlighted (absolute t > 16) (Figure 2C). Notably, the pyrimidine metabolism and purine metabolism pathways exhibited considerable upregulation, ranking prominently among all pathways (Figure 2C), which was further validated utilizing The Cancer Genome Atlas (TCGA) dataset of BC and normal breast tissues (Figure S2C). It is worth noting that both pyrimidine and purine metabolism pathways showed prominent upregulation in tumor epithelial cells as well as fibroblasts, myeloid cells, and T cells, while significant downregulation in tumor endothelial cells compared to cells in normal tissues (Figure 2E). We also characterized global metabolic changes in tumor epithelial cells compared to normal epithelial cells by sorting the metabolic pathways by rank order, illustrating marked changes in both of the nucleotide metabolism pathways including purine and pyrimidine (Figures S2D, S2E, and 2D). Furthermore, we examined the expression levels of pivotal metabolic genes encoding key enzymes in nucleotide metabolism pathways. As illustrated in Figure 2F, in comparison to normal epithelial cells, BC cells exhibited enhanced the utilization of inosine and uridine as ribose carbon sources for DNA and RNA synthesis. Together, these data demonstrate the critical role of purine and pyrimidine metabolism in BC at the single-cell level. Moreover, we further confirmed our findings using an independent validation scRNA-seq dataset of BC, which comprised approximately 0.2 billion unique transcripts from 6 breast tumor samples and 4 adjacent nonmalignant breast tissues for comparative analysis (Figures S3A–S3H).^4^^,^^34^

Given the critical roles of nucleosides in activating immune cells to regulate antitumor immunity as recently reported,^31^^,^^32^ we further explored the role of nucleotide metabolism in the tumor microenvironment of different subtypes of BC. Utilizing a previously published scRNA-seq,^4^ including 64,738 cells originating from nineteen primary breast tumors including 7 TNBCs, 8 HR+/HER2-and 4 HER2+, we observed differences in purine and pyrimidine metabolism between TNBC and non-TNBC subjects (Figures 2G, 2H, S4A, and S4B), as well as in a three-group comparison of HR+/HER2-, HER2+ and TNBC subjects (Figures S5A and S5B), illustrating higher activating levels of nucleotide metabolism of TNBC in various cell types including tumor epithelial cells and T cells. Considering that TNBC is of higher malignancy and worse prognosis and a lower proportion of patients with TNBC show response to immunotherapy, we sought to investigate the correlation between nucleotide metabolism in tumor epithelial cells and the activity of immune cells in the tumor microenvironment. Our result showed that higher purine metabolism in tumor epithelial cells is strongly associated with markers of Treg activation (*CCR8*, *FOXP3,* and *IL2RA*) and levels of *ADORA2A*, encoding the well-known purine receptor A2AR, in Tregs (Figures 2I, S4C, and S4D; Table S3). We further analyzed the expression of A2AR in various T cell subtypes, illustrating significantly elevated levels of A2AR in Tregs of TNBC compared to non-TNBC (Figure 2J). We also identified upregulated activity of Tregs in TNBC compared to non-TNBC (Figure 2K), suggesting that purine metabolism may enhance Treg activity via A2AR in TNBC. Collectively, these evidence suggests a role of nucleotide metabolism in the regulation of Treg activity and antitumor immunity, which may ultimately result in distinct prognoses of different subtypes of BC.

### Patients with breast cancer illustrate distinct profiles of plasma metabolites involved in pathways of nucleotide metabolism

**Figure 3:**
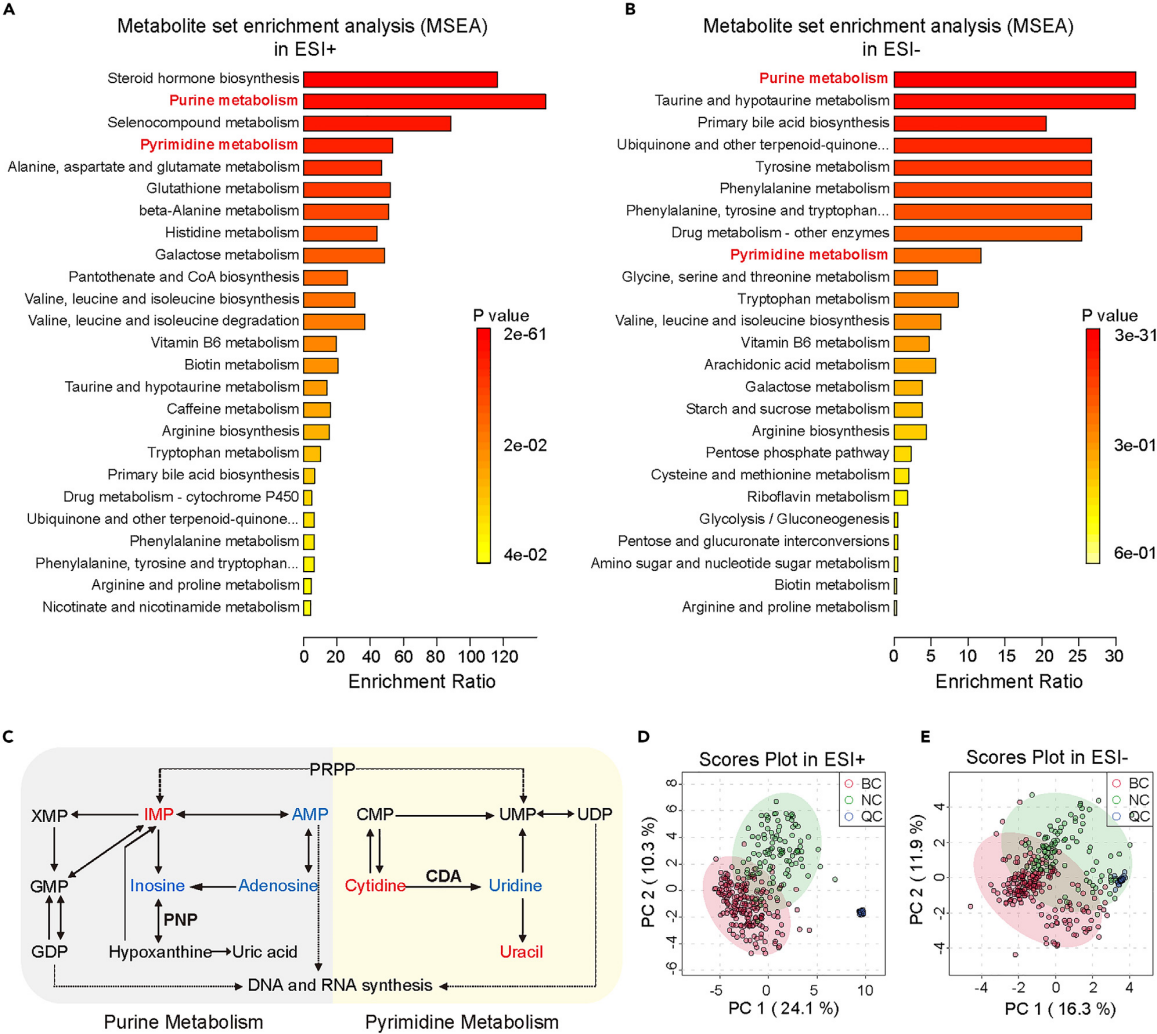
Overview of untargeted plasma metabolomic analysis in the exploratory study (A and B) Pathway enrichment analysis for metabolites is significantly different between BC and NC groups in ESI+ mode (A) and ESI- mode (B) of untargeted plasma metabolomic analysis. The *p* values presented were adjusted for multiple comparisons using the Benjamini-Hochberg procedure (FDR). (C) Dysregulated metabolites involved in purine metabolism and pyrimidine metabolism in tumor epithelial cells. T-test analysis was performed and the FDR-adjusted *p* value cutoff was set to 0.05. Up-regulated metabolites are marked in red, while down-regulated metabolites are marked in blue. (D and E) PLS-DA score plots for untargeted metabolomics data from the exploratory cohort in ESI+ mode (D) and ESI- mode (E).

To characterize plasma metabolites in patients with BC, we examined peripheral blood plasma samples from 200 BC to 100 NC through LC-DDA-MS-based untargeted metabolomics analysis (Figure S1A; Table S4). Raw MS data were processed using MS-DIAL software (version 4.70) for spectra deconvolution, peak detection, adduct identification, and alignment among samples. The identification score threshold was set at 80% to ensure robust identification. We performed metabolite set enrichment analysis (MSEA) using differential plasma metabolite features between NC and BC, showing an obvious enrichment in pathways of nucleotide metabolism (Figures 3A and 3B). Levels of metabolites involved in purine and pyrimidine metabolism pathways were then compared between NC and BC respectively, and our data showed that most of the metabolites we detected in these two pathways exhibited massive changes (Figure 3C).

### A highly accurate model for the early detection of breast cancer is established via machine learning-based feature selection and modeling

**Figure 4:**
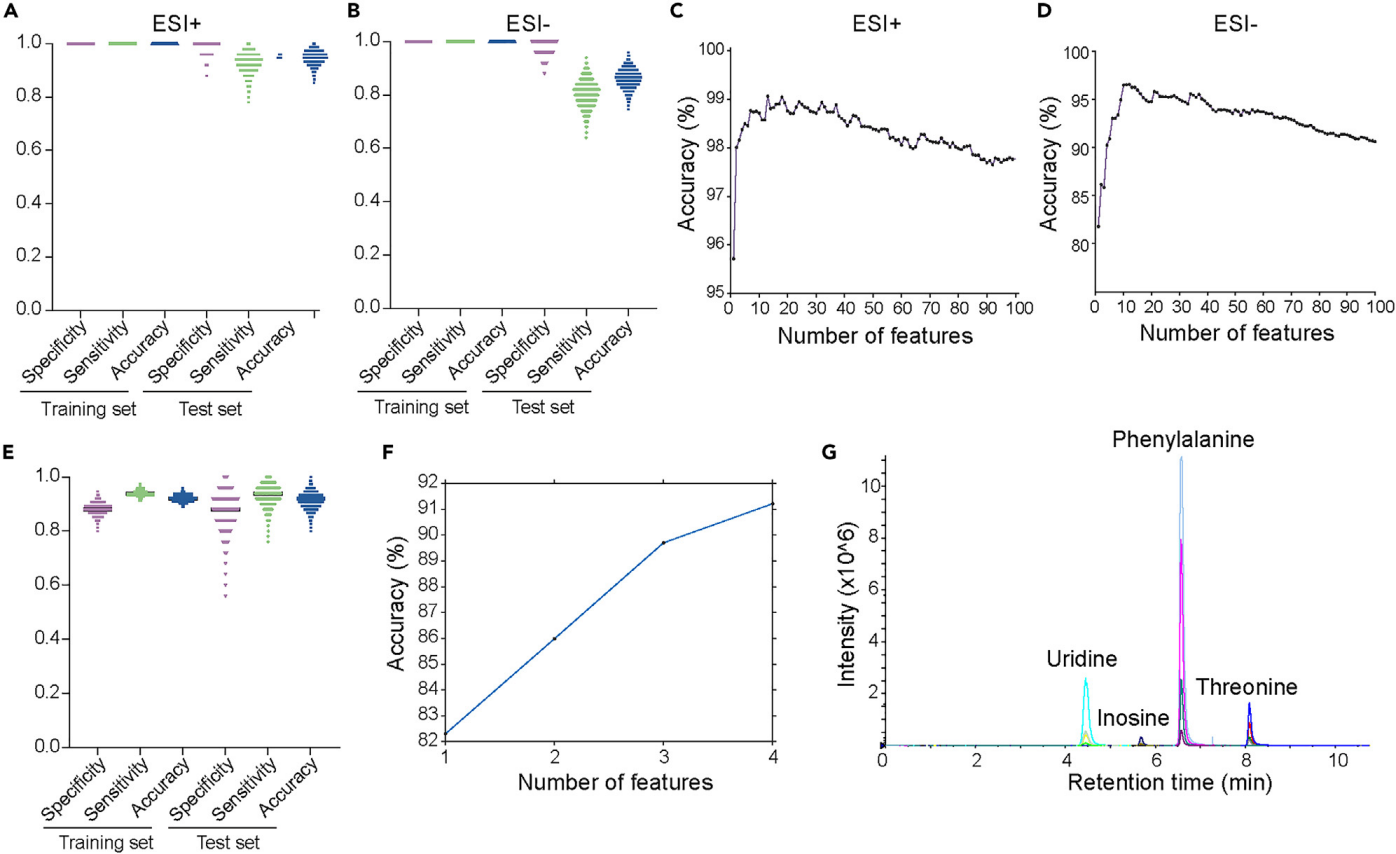
ML-based feature selection and development of the HILIC-MRM-MS targeted method for BC detection (A and B) Classification performance metrics of the SVM-based BC detection approach on the training and test set in ESI+ mode (A) and ESI- mode (B) (data presented as means ± SD; *N* = 2000 iterations, with each dot representing data for one iteration of SVM evaluation). (C and D) The mean classification accuracy of the SVM model using the top 100 selected features in ESI+ mode (C) and ESI- mode (D). (E) Classification performance metrics of the SVM-based approach for BC detection on the training and test sets with four selected features (data presented as means ± SD; *N* = 2000 iterations, with each dot representing data for one iteration of the SVM evaluation). (F) Mean classification accuracy of the SVM-based approach for the BC detection of the SVM model utilizing the four selected features. (G) Extracted ion chromatograms depicting the quantification of the four selected metabolites using the MRM assay in a single 19-min LC-MS run.

To further select robust plasma metabolic features to characterize BC, features without matched MS/MS spectra were excluded from subsequent analysis. A total of 479 features were identified in the positive mode of electrospray ionization (ESI), while 269 features were identified in the negative mode. To remove unwanted variations related to signal drift/attenuation at the feature level, the alignment results were subjected to statTarget analysis with the quality control-based random forest signal correction algorithm (QC-RFSC) before obtaining the final dataset for subsequent statistical analysis and feature selection.^35^ As shown in the Partial Least Squares-Discriminant Analysis (PLS-DA) score plots (Figures 3D and 3E), BC was relatively well separated from NC, while the quality control samples (QC) were well clustered in both ESI+ (Figure 3D) and ESI- (Figure 3E) ion modes, indicating that the system is stable and reliable. Cross-validation and *permutation tests were performed to ensure the PLS-DA models were not overfitting* (Figures S6A–S6D). As shown in Figures 1B and S1A, we used a dataset comprising all 300 samples analyzed through untargeted metabolomics in the exploratory cohort to develop SVM algorithm-based ML models and assess their classification performance. The procedure was iterated randomly for 2000 times of 4-fold cross-validation. Each feature was assigned a squared mean weight based on its contribution to the classification of the BC and NC groups. The performance of the classification model was evaluated by 2000 iterations, demonstrating a result that the mean training accuracy was 100.0% (95% CI, 100.00%–100.00%), and the mean test accuracy was 96.38% (95% CI, 96.29%–96.47%) with a mean specificity of 98.62% (95% CI, 98.52%–98.72%) and a mean sensitivity of 95.26% (95.14%–95.39%) in the data from ESI+ mode (Figure 4A; Table S5), and, the mean test accuracy was 86.21% (95% CI, 86.06%–86.35%), with a mean specificity of 97.59% (95% CI, 97.46%–97.71%) and a mean sensitivity of 80.52% (80.30%–80.73%) (Figure 4B; Table S5) in the data from ESI- mode. These findings indicate that the integration of plasma metabolomics and SVM presents a promising strategy for BC detection.

To identify the most relevant set of features and remove irrelevant ones for targeted validation, attributive weights for all features were determined by the classification model, reflecting their importance in the SVM-based classification process. Based on these weights, we progressively selected the top 100 most important features from the highest-ranking features to construct predictive models. Each iteration of the top-N feature selection involved 500 iterations of 4-fold cross-validation to ensure reliable predictions. After 50,000 iterations, the mean accuracies for each predictive model (*n* = 100) in feature selection were calculated (Figures 4C and 4D). To achieve improved classification performance with a relatively small number of features, a total of 14 metabolites including 5 metabolites in the ESI+ mode and 10 metabolites in the ESI- mode (1 metabolite overlapped) were selected as candidate features. Subsequently, these candidate features with unmatched MS/MS to commercially available chemical standards were filtered, and chromatographic peak shapes were evaluated to ensure their reliability and suitability for targeted quantification analysis (Figures S7A–S7E). Ultimately, a panel of 4 metabolites including inosine, uridine, phenylalanine, and threonine, was selected (Table S6) and a new four-feature model was constructed by combining untargeted data from these four features selected from both positive and negative ion modes. The mean classification accuracy of the four-feature model on the testing set was 91.32% (95% CI, 91.19%–91.45%) with a mean specificity of 87.69% (95% CI, 87.40%–87.98%) and a mean sensitivity of 93.13% (95% CI, 92.97%–93.30%) (Figures 4E and 4F). These results demonstrate that the selected four features contain sufficient information to effectively discriminate BC from NC, and contribute to the development of targeted approaches for the early detection of BC.

### Targeted MRM analysis of a four-metabolite panel demonstrates the robustness and generalization of our predictive model in large independent cohorts

We then developed a hydrophilic interaction liquid chromatography-multiple reaction monitoring (HILIC-MRM) targeted quantitative assay. Stable isotope labeled-internal standards (SIL-IS) of each target metabolite were utilized to compensate for matrix effect as well as errors in the extraction and sample preparation procedures. Through optimizing multiple parameters including LC elution gradient, mobile phase additives, reconstitution conditions, transition selection, CE (collision energy), and DP (declustering potential), and so forth, an assay with satisfactory chromatographic peak shape and higher mass sensitivity was established that could quantify the 4 selected metabolite markers in a single 19-min LC-MRM-MS run of ESI+ ion mode (Figures 4G and S8A–S8C; Tables S7 and S8). Extracted-ion chromatograms (XIC) of the four selected metabolites in MRM results before and after method optimization are shown in Figures S9A and S9B, illustrating a significant enhancement in the chromatographic peak shape and mass spectral response after optimization to ensure quantitative accuracy and precision. Detailed information of the standard curves (Figures S10A–S10D) including the coefficient of determination values, lower limits of detection (LLOD), lower limits of quantification (LLOQ), and linear ranges of four selected metabolites were listed in Tables S9 and S10.

**Figure 5:**
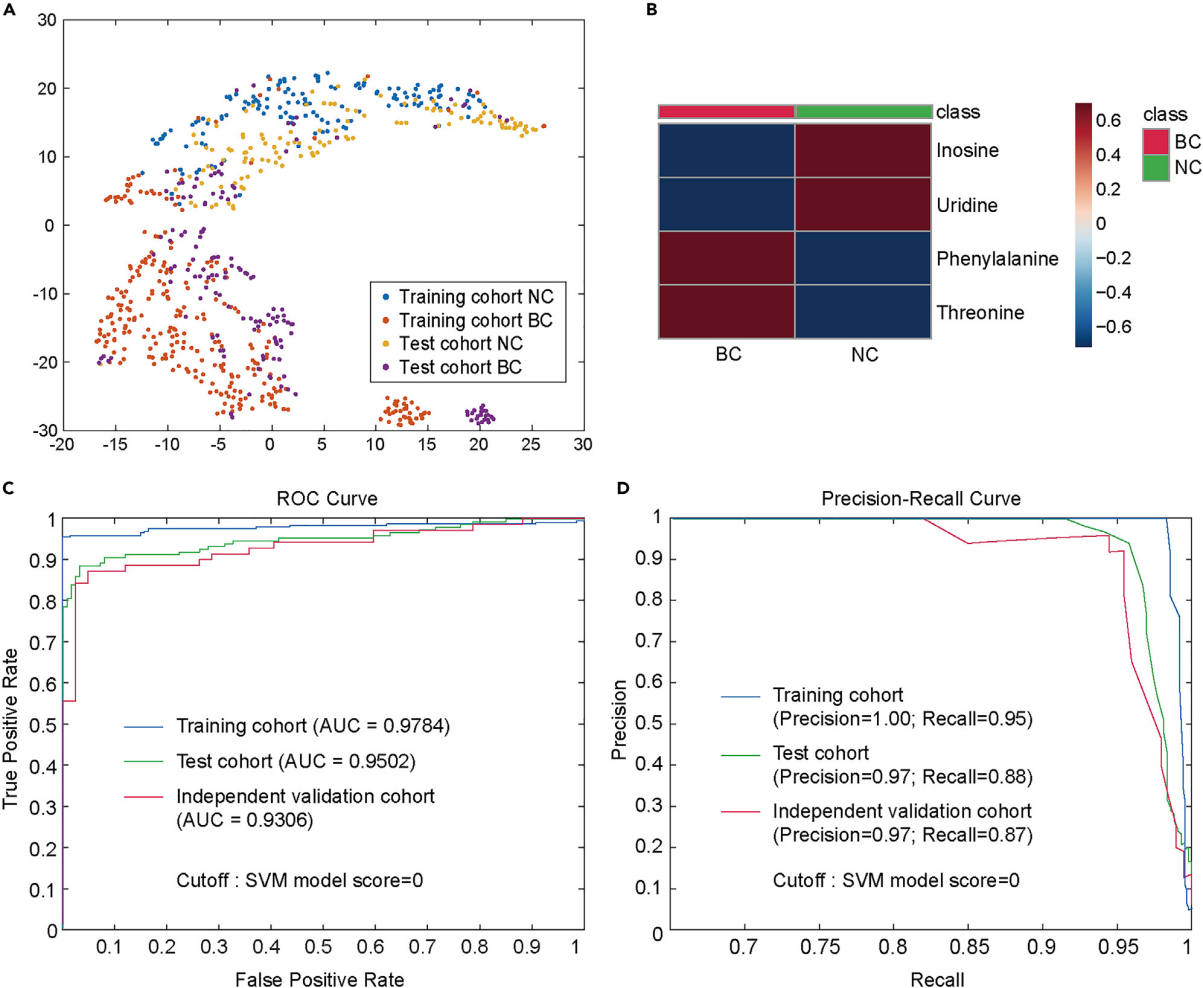
SVM-based diagnostic modeling and performance evaluation in the validation study (A) t-SNE visualization of the MRM datasets from the training cohort and test cohort in the validation study. Different datasets are color-coded (blue, training cohort NC; red, training cohort BC; yellow, test cohort NC; purple, test cohort BC). (B) Heatmaps of average quantities for the four selected metabolites in the targeted MRM assay. (C) ROC curves of the training cohort (blue), test cohort (green) and independent validation cohort (red) processed by the SVM-based diagnostic model. (D) Precision-Recall curves for the training cohort (blue), test cohort (green) and independent validation cohort (red) processed by the SVM-based diagnostic model.

To construct and validate an ML-based detection model based on targeted analytic data in a validation study (Figure 1C), samples from a total of 699 participants were analyzed using the absolute quantitative targeted assay for the first batch. Among them, 423 samples were included in the training cohort for diagnostic modeling, and 276 samples were assigned to the test cohort for evaluating the model’s performance (Figure S1A; Table S4). The quantitative data of the four selected metabolites in both the training and test cohorts could be effectively clustered using t-SNE (t-distributed stochastic neighbor embedding) (Figure 5A), and average quantities are shown in Figure 5B. Then the absolute concentrations from the training cohort were utilized to establish an SVM-based detection model, which was subsequently evaluated on the test cohort in the validation study (c = 5 in SVM). For the training cohort, the classification model demonstrated impressive discriminatory performance, achieving an accuracy of 94.09% and an area under the ROC curve (AUC) of 0.9784. The model exhibited a specificity of 90.71% and a sensitivity of 95.76%. In the test cohort, the prediction accuracy reached 90.94% with an AUC of 0.9502, accompanied by a specificity of 92.86% and a sensitivity of 89.33% (Figures 5C and 5D; Table S11). These findings provide compelling evidence for the reliability of our workflow of feature selection, and the significance of the four-metabolite-marker panel in BC detection.

To further validate the performance of the detection model, an independent validation cohort consisting of 112 plasma samples was utilized as an external validation test dataset for additional evaluation. Among these samples, 42 control samples were prospectively recruited, while 70 BC samples were independently obtained from another hospital (Figure S1A; Table S4). The classification accuracy of our model in this external validation test set reached 89.29%, with an AUC of 0.9306. The model demonstrated a specificity of 92.86% and a sensitivity of 87.14% (Figures 5C and 5D; Table S11). These results highlight the strong discriminatory capability of our model in distinguishing BC cases from healthy controls.

### Nucleoside markers are of predictive value for neoadjuvant chemotherapy outcomes in patients with triple-negative breast cancer

**Figure 6:**
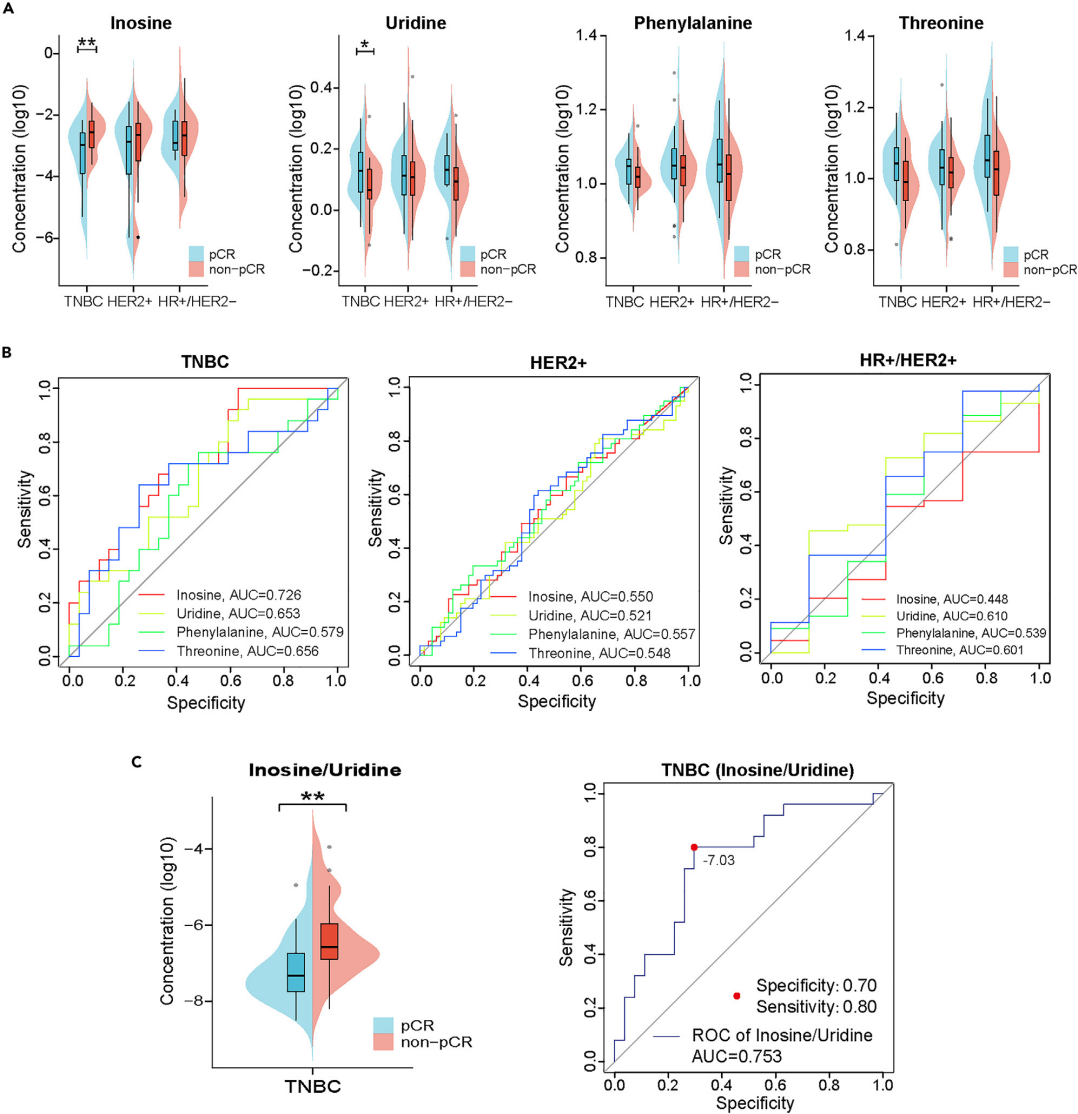
Predictive values of four selected markers for NAC outcomes in patients with TNBC (A) The bean plot showing differences in the four metabolite markers between the pCR and non-pCR group among three subtypes of BC (∗*p* < 0.05, ∗∗*p* < 0.01, ∗∗∗*p* < 0.001, ∗∗∗∗*p* < 0.0001). (B) ROC curves illustrating the predictive performance of the four metabolites among three subtypes of BC. (C) The bean plot and the ROC curve illustrate the predictive performance of the pairwise ratio of inosine and uridine between the pCR and non-pCR groups within patients with TNBC.

For patients with BC, choosing a proper therapeutic diagram is critical for achieving a better prognosis. There are still no effective predictive biomarkers for the response to NAC in TNBC at present.^2^^,^^17^^,^^18^ Therefore, we further investigated the predictive potential of the 4 selected markers for NAC outcomes. As shown in Figure 6A, among the four metabolite markers, inosine and uridine showed marked differences between the pathological complete response (pCR) (*n* = 27) and non-pCR group (*n* = 25) within patients with TNBC, while no significant difference existed in the other two subtypes of BC. ROC curves were also depicted to illustrate the predictive performance of the four metabolites (Figure 6B). Furthermore, using the pairwise ratio of inosine and uridine, the predictive performance showed further improvement with an AUC of 0.753 (Figure 6C). These data strongly suggest that inosine and the inosine/uridine ratio are of great potential to be applied as predictive markers for NAC outcomes in patients with TNBC to guide clinical decisions of therapeutic diagrams.

## Discussion

According to our data, purine metabolism, pyrimidine metabolism, phenylalanine metabolism, and glycine serine and threonine metabolism all demonstrated significant dysregulation in BC cells (Figures 2D, S2D, S2E, and S3F–S3H). These analyses demonstrated substantial alterations in the metabolic pathways involving the four selected metabolites in BC cells. Inosine and uridine play vital roles in nucleotide salvage pathways, with inosine acting as a central intermediate in purine metabolism, and uridine serving as the primary substrate in pyrimidine metabolism.^31^ Our tissue scRNA-seq analysis revealed significant upregulation of the purine and pyrimidine metabolic pathways in cancer, which is consistent with previous consensus that maximizing the use of nucleoside salvage pathways benefits cancer cell growth by saving energy and biomass for other anabolic processes.^31^ Consistent with previous findings that cancer cells can harness extracellular inosine and uridine as energy sources to sustain rapid proliferation,^36^^,^^37^^,^^38^^,^^39^^,^^40^ our study revealed reduced levels of inosine and uridine in the plasma of patients with BC. This suggests that mammary tumor cells may augment the acquisition and utilization of inosine and uridine from plasma, leading to a decline in these nucleosides.

Recent studies have revealed that abnormal nucleotide metabolism not only accelerates tumor development but also impacts the tumor microenvironment (TME) to limit immune responses.^41^^,^^42^^,^^43^^,^^44^ In our study, single-cell sequencing analysis revealed a strong correlation between the nucleotide metabolism of breast tumor cells and the activation of Treg cells, and this association may be linked to the adenosine receptor, A2AR (Figure 2I; Table S3). Notably, we observed a significant difference in A2AR receptor expression between TNBC and other BC subtypes, particularly evident in Treg cells (Figure 2J), potentially contributing to the unfavorable prognosis associated with TNBC. In recent years, the role of Treg cells in BC progression and metastasis has attracted increased attention. Studies have indicated that the accumulation of Treg cells in TNBC can boost immune disruption,^45^^,^^46^ and the abundance of Treg cells may serve as a predictive biomarker for NAC outcomes in TNBC.^47^ These findings align with our results from scRNA-seq and plasma metabolomics. Furthermore, our study suggests that enhanced purine metabolism in TNBC may upregulate Treg activity via A2AR, contributing to immune suppression and a worse prognosis for patients with TNBC.

In our data of plasma metabolomics, the downregulation of inosine, a potent A2AR agonist, is associated with pCR after NAC in TNBC. Consequently, we hypothesize that reduced inosine levels in the plasma may inhibit Treg activation in the TME and promote the activation of anti-tumor immune responses, offering a better chance to achieve pCR for patients with TNBC undergoing NAC. In light of these findings, targeting inosine metabolism holds promise as a promising approach to enhance the tumor microenvironment for cancer immunotherapy.

Additionally, although research on plasma or serum amino acid levels is relatively scarce (and often limited in scale by small sample numbers), the potential of plasma-free amino acid (PFAA) profiles as biomarkers of malignancy has attracted significant attention.^48^^,^^49^ Importantly, consistent with our findings, several studies have reported elevated plasma levels of phenylalanine and threonine in patients with BC.^50^^,^^51^ However, the mechanisms driving changes in PFAA profiles among tumor patients remain contentious and poorly understood. It has been suggested that, apart from their partial origin in tumor-related protein metabolism, these changes may predominantly stem from altered metabolism of host skeletal muscle and liver proteins.^52^^,^^53^

The integration of artificial intelligence algorithms into biomedical data analysis has emerged as a potent strategy for augmenting model efficiency and predictive capabilities, particularly in the diagnosis, progression assessment, and prognosis prediction of diseases.^54^^,^^55^^,^^56^^,^^57^ Within BC research, the amalgamation of artificial intelligence and metabolomics to establish a reliable and efficient blood-based early detection model has posed many challenges. Our study depended on the judicious selection of discriminatory and pivotal differential features from the untargeted metabolome. The utilization of the supervised classification algorithm SVM for feature selection, coupled with the iterative assignment of average importance weights to each feature, facilitated the creation of the SVM-based target prediction model. Moreover, the implementation of an absolute quantification method based on the SIL-IS calibration curve yielded precise concentrations of the four targeted metabolites, thereby enhancing the precision and consistency of our model. As illustrated in Table S6, the four compounds exhibit substantial variations in terms of acid-base ionization (as determined by pKa values), a factor that significantly influences retention, selectivity, and peak shape within a chromatographic separation.^58^^,^^59^^,^^60^ Simultaneously, the marked dissimilarities in their endogenous concentrations exacerbate the challenges associated with their concurrent quantification (Table S6).

In this study, we have effectively developed a model for the early detection of BC by the integration of machine learning and absolute quantitative metabolomics. This approach with only four metabolites, demonstrated an excellent ability to provide consistent and dependable results in multiple cohorts, while also presenting cost-effective and swift processing capabilities. The design of our study meticulously ensured the incorporation of more than 70% of early-stage BC samples (stage 0-II) within the clinical cohort. This deliberate selection aimed to authenticate the efficacy of our detection model for early BC diagnosis. We believe that the strategic inclusion of samples sourced from different origins and diverse batches enhances the reliability of our model. Looking ahead, we anticipate that the rapid evolution of metabolomics and artificial intelligence technology will lead to the discovery of numerous previously unidentified metabolic features and improve the analysis of complex omics data. Furthermore, we expect to witness increased integration of multi-omics data, encompassing genomics, transcriptomics, proteomics, and metabolomics, to gain a deeper understanding of breast cancer biology. Additionally, advancements in AI techniques will empower the development of more accurate and individualized diagnostic and treatment approaches.

In summary, our joint analysis of single-cell transcriptome and metabolomics revealed a pivotal role of nucleotide metabolism in tumor immunity and illustrated varied levels of nucleotide metabolism in different subtypes of BC, shedding light on the regulatory mechanisms underlying the metabolite changes observed in BC. Our study adeptly leveraged the SVM-based ML algorithm, in conjunction with untargeted and absolute quantitative targeted metabolomics methodologies, to establish an innovative diagnostic panel comprising only four metabolic markers. This non-invasive, rapid, and cost-effective diagnostic approach exhibits high specificity and sensitivity, holding significant potential for early BC detection and diagnosis. Moreover, purine analogs including inosine and uridine showed good predictive capability for NAC response in patients with TNBC, providing blood-based markers to facilitate the choice of therapeutic regimen and improvement of prognosis.

### Limitations of the study

Several limitations of our study should be acknowledged. The limited diversity of identified metabolites in current metabolomics studies stems from challenges in detecting metabolites with varying concentrations and structural complexities, as well as limitations in sensitivity, specificity, sample processing, and data analysis techniques. These factors collectively hinder comprehensive metabolite identification and interpretation. At present, the types of metabolites that can be identified in untargeted metabolomics are still limited, and more valuable potential metabolic diagnostic markers have yet to be discovered. This approach requires extensive analysis and clinical validation in larger multicenter, multiethnic cohorts before it can be formally used as an early detection tool for BC. In addition, the great potential of inosine and the inosine/uridine ratio as predictive markers for NAC outcomes in TNBC patients’ needs validation in larger sample sizes.

## STAR★Methods

### Key resources table

**Table:** 

REAGENT or RESOURCE	SOURCE	IDENTIFIER
**Biological samples**		

Plasma	Peking University People’s Hospital, Peking University Shenzhen Hospital, PKUCare Health Management Center of Peking University Healthcare Group	2021PHB387-001
Tumors tissues and adjacent tissues	Peking University People’s Hospital	2021PHB387-001

**Chemicals, peptides, and recombinant proteins**		

Methanol HPLC grade	Thermo Fisher	A452-4; CAS: 67-56-1
Acetonitrile LC-MS grade	Thermo Fisher	A955-4; CAS: 75-05-8
Formic acid additive for LC-MS	Sigma-Aldrich	F0507-100ML; CAS: 64-18-6
Ammonium acetate solution additive for LC-MS	Sigma-Aldrich	A2706-100ML; CAS: 631-61-8
Inosine	Sigma-Aldrich	CAS: 58-63-9
Uridine	Sigma-Aldrich	CAS: 58-96-8
Phenylalanine	Sigma-Aldrich	CAS: 63-91-2
Threonine	Sigma-Aldrich	CAS: 72-19-5
^13^C5-inosine	ISOREAG	Cat.No. IR-25780
^13^C5-uridine	ISOREAG	CAS: 159496-16-9
d8-Phenylalanine	ISOREAG	CAS: 17942-32-4
^15^N-threonine	ISOREAG	CAS: 80681-09-0

**Deposited data**		

Single-cell RNA-seq data	This paper	GEO: GSE268662
Metabolomics data	This paper	MassIVE: MSV000093325
Raw data from Figures 4A–4E	This paper	Mendeley Data: https://doi.org/10.17632/wcxw6m2hhv.1
original code	This paper	Zenodo: https://doi.org/10.5281/zenodo.10078185

**Software and algorithms**		

MS-DIAL (version 4.70)	Tsugawa et al.^61^	https://systemsomicslab.github.io/compms/msdial/main.html
MATLAB (version 2022b)	MathWorks	https://www.mathworks.com/products/matlab.html
R (version 4.0.3)	R Core Team (2020)	https://cran.r-project.org/
MetaboAnalyst 6.0	Pang et al.^62^	https://www.metaboanalyst.ca/faces/home.xhtml
Graphpad Prism (version 9.0.0)	Graphpad	https://www.graphpad.com/scientific-software/prism/
SPSS (version 26.0.0)	IBM	https://www.ibm.com/products/spss-statistics
R package DoubletFinder (version 2.0.3)	McGinnis et al.^63^	https://www.sciencedirect.com/science/article/pii/S2405471219300730
R package Limma (version 3.48.3)	Ritchie et al.^64^	https://bioinf.wehi.edu.au/limma/
Subread package (version 1.6.4)	Liao et al.^65^	https://subread.sourceforge.net/
statTarget	Luan et al.^35^	https://stattarget.github.io/
STAR (version 2.5.1b)	Dobin et al.^66^	https://doi.org/10.1093/bioinformatics/bts635
Cell Ranger	10x Genomics	https://www.10xgenomics.com/
MultiQuant MD (version 3.0.3)	SCIEX	https://sciex.com/products/software/multiquant-software
liblinear	Fan et al.^67^	http://www.csie.ntu.edu.tw/∼cjlin/liblinear

### Resource availability

#### Lead contact

Further information and requests for resources should be directed to and will be fulfilled by the lead contact Professor Yuxin Yin (yinyuxin@bjmu.edu.cn).

#### Materials availability

This study did not generate new unique reagents.

#### Data and code availability

• Single-cell RNA-seq data have been deposited at GEO and are publicly available as of the date of publication. Accession numbers are listed in the key resources table. Metabolomics data have been deposited at MassIVE and are publicly available as of the date of publication. Accession numbers are listed in the key resources table. Raw data from Figures 4A–4E have been deposited at Mendeley and are publicly available as of the date of publication. The DOI is also listed in the key resources table.
• All original code has been deposited at Zenodo and is publicly available as of the date of publication. DOIs are listed in the key resources table.
• Any additional information required to reanalyze the data reported in this paper is available from the lead contact upon request.

### Experimental model and study participant details

For single-cell transcriptome sequencing analysis, tumors tissues and adjacent tissues from five BC patients were collected at Peking University People’s Hospital. A total of 1111 all female participants were included in the plasma metabolomic analysis, comprising 703 BC patients and 408 NC. The NC samples for the exploratory study were recruited from the Peking University People’s Hospital, while those for the validation study were enrolled from the PKUCare Health Management Center of Peking University Healthcare Group. The BC samples used in the exploratory study, training cohort, and test cohort of the validation study were collected from Peking University People’s Hospital. The BC samples utilized for the independent validation cohort in the validation study were obtained from Peking University Shenzhen Hospital.

All samples were obtained from female Chinese participants aged 24 to 82 years. In each metabolomics cohort, early-stage BC samples (stage 0-II) accounts for more than 70% of all BC samples. The clinicopathological characteristics of the subjects enrolled in the exploratory study and the validation study are summarized in Table S4. This study was approved by the Ethics Committee of Peking University People’s Hospital (2021PHB387-001), and written informed consent was obtained from all participants in accordance with the ethical standards set forth in the Declaration of Helsinki.

### Method details

#### Inclusion criteria

For the BC patients, the inclusion criteria were as follows: pathologically confirmed BC; no anti-tumor therapy such as neoadjuvant chemotherapy and targeted therapy before surgery; no history of other malignant tumors; and no common metabolic diseases such as diabetes or other severe systemic disease. The NC groups were meticulously selected to match the BC groups in terms of age and sex, with an additional criterion of no history of cancer or systemic diseases.

#### Preparation of single cell suspensions

Freshly collected tissue samples were immediately washed with RPMI1640 medium (Gibco, Life Technologies) and dissociated using a Multi-tissue dissociation kit 2 (Miltenyi 130-095-929). The addition of DNase was dependent on the viscosity of the homogenate. Subsequently, cell counts and viability were determined using a fluorescence Cell Analyzer (Countstar Rigel S2) with AO/PI reagent, following the removal of erythrocytes (Miltenyi 130-094-183). The decision to perform debris and dead cell removal was made based on experimental requirements (Miltenyi 130-109-398/130-090-101). Finally, the freshly isolated cells were again washed twice with medium and resuspended at a concentration of 1×10^6^ cells per mL in 1×PBS and 0.04% bovine serum albumin (BSA) (Thermo Fisher Scientific).

#### Single cell RNA-seq library construction and sequencing

Single-cell RNA-Seq libraries were prepared using the SeekOne MM Single Cell 3′ library preparation kit (SeekGene Catalog No.). Briefly, an appropriate number of cells were loaded into the flow channel of the SeekOne MM chip, which contained 170,000 microwells, and allowed to settle in the microwells by gravity. After removing unsettled cells, Cell Barcoded Magnetic Beads (CBBs) was pipetted into the flow channel and also allowed to settle in the microwells with the assistance of a magnetic field.

Next, excess CBBs were removed, and the cells in the MM chip were lysed to release RNA, which was captured by the CBB in the same microwell. Subsequently, all CBBs were collected, and reverse transcription was performed at 37°C for 30 min to label the cDNA with the cell barcode on the beads. Exonuclease I treatment was then carried out to remove unused primers on the CBBs.

Following that, the barcoded cDNA on the CBBs was hybridized with a random primer that had the 2 SeqPrimer sequence on the 5′ end and could extend to form the second strand DNA with the cell barcode on the 3′ end. The resulting second strand DNA was denatured off the CBBs, purified, and amplified in a PCR reaction. The amplified cDNA product was subsequently purified to remove unwanted fragments and added to full-length sequencing adapters and sample indices by indexed PCR. The indexed sequencing libraries were cleaned up with SPRI beads, quantified using quantitative PCR (KAPA Biosystems KK4824), and then sequenced on the Illumina NovaSeq 6000 platform with PE150 read length or the DNBSEQ-T7 platform with PE150 read length.

#### Sequencing data quality control

Fastp (v0.20.1) was employed to perform primer sequence trimming and removal of low-quality bases from the raw reads,^68^ as well as to collect basic statistics. The specific parameters utilized are summarized as follows: (i) A 4 bp sliding window was applied, starting from the front (5′) end. If the mean quality of bases within the window fell was less than 10, those bases within the window and subsequent bases were discarded, and the analysis for that read was concluded. Additionally, any leading N bases were trimmed using the parameters --cut_front --cut_front_window_size 4 --cut_front_mean_quality 10. (ii) A 1 bp sliding window was used, moving from the tail (3′) end to front. If the mean quality of bases within the window dropped below 3, those bases within the window were removed, and the window continued moving until the last base. Any trailing N bases were trimmed accordingly, employing a method similar to Trimmomatic’s TRAILING option (--cut_tail --cut_tail_window_size 1 --cut_tail_mean_quality 3). (iii) For paired-end (PE) data, the automatic adapter detection feature was enabled (detect_adapter_for_pe). (iv) Reads shorter than 60 bp, following the trimming steps, were discarded (--length_required 60). The resulting adjusted reads obtained after the trimming processes were used for subsequent analyses.

#### Single cell RNA-seq data processing

The Seekoul Tools pipeline was utilized to process the reads and generate the transcript expression matrix. Initially, the cell barcodes and Unique Molecular Identifier (UMI) sequences were extracted based on a defined pattern that localized the barcode, linker, and UMI within each read. The extracted barcodes were corrected using a whitelist, and the corrected barcode, along with the UMI, were placed in the header of their respective reads. Next, the reads were aligned to the reference genomes using STAR 2.5.1b.^66^ Subsequently, reads containing barcode and UMI information were assigned to the transcriptome using featureCounts from the Subread package 1.6.4,^65^ with the default parameters except for "-s 1-fracOverlap 0.5". This step resulted in a raw UMI count matrix based on barcodes and transcripts, similar to the raw_feature_bc_matrix output of Cell Ranger.^69^

To filter the raw UMI count matrix and obtain the filtered_feature_bc_matrix containing only cells, a cell-calling algorithm was employed. This algorithm resembled those used by Cell Ranger and EmptyDrops,^70^ involving two key steps: (i) the algorithm employed a cutoff based on the total UMI counts of each barcode to identify cells. This step identified the primary mode of high RNA content cells; (ii) the algorithm utilized the RNA profile of each remaining barcode to determine whether it represented an "empty" partition or contained a cell. This step captured low RNA content cells with total UMI counts that might be similar to empty wells.

#### scRNA-seq quality control, batch effect correction and clustering

Cells were removed that had either less than 200 or more than 5000 expressed genes. Furthermore, we discarded cells with mitochondrial content higher than 25%. Additionally, the DoubletFinder R package (version 2.0.3) was applied for each library separately to identify potential doublets and cells predicted to be doublets were excluded.^63^ After quality control, a total of 51549 single cells from 9 libraries were retained for downstream analysis.

For each library, we used Seurat (version 4.1.0) to first normalize expression matrices by function NormalizeData, and the top 2000 most variable genes were identified by the “vst” method in FindVariableFeatures. Individual data were further integrated to remove batch effects using an anchor-based method.^71^

For the integrated data, we used ScaleData to scale z-scores for each variable gene and performed principal component analysis on the scaled data. The first 15 principal components and a resolution 0.5 were used with the FindClusters function to generate 20 cell clusters. For visualization, the dimensionality of the integrated data was further reduced using the Uniform Manifold Approximation and Projection (UMAP) by the Seurat function RunUMAP.

#### Differential gene expression analysis and cluster annotation

To identify cell cluster-specific differentially expressed genes (DEGs), two-sided unpaired Wilcoxon tests were performed on all the expressed genes (expressed in at least 10% of cells in either cluster of cells) by using the FindAllMarkers function. Genes with a Bonferroni-corrected *p* value < 0.05 and an average log-fold change >0.25 were considered differentially expressed. Clusters were annotated by comparing DEGs with markers previously associated with T cells (*CD247*, *CD4*, *CD8A*), B cells (*MS4A1*, *CD79A*), plasma (*JCHAIN*), endothelial cells (*FLT1*, *EMCN*), epithelial cells (*EPCAM*, *KRT18*), fibroblasts (*PDGFRA*, *COL3A1*), mast cells (*SLC18A2*, *KIT*), myeloid cells (*CD14*, *LYZ*, *S100A8*) and pericytes (*ACTA2*, *TAGLN*), CD4^+^ T cells (*CD4*), CD8^+^ T cells (*CD8A*), NK cells (*NCAM1*), Treg cells (*FOXP3*), proliferating T cells (*MKI67*). Marker genes were visualized on UMAP projections using log-normalized counts. To generate bubble plots of the marker genes, the average expression level of each gene was calculated for each cluster/group and then normalized by mean and standard deviation (z-scores).

#### Copy number alterations detection

We inferred copy number alterations of epithelial cells from tumor tissues by InferCNV using single-cell transcriptomic profiles.^33^ Single cell RNA-seq data of epithelial cells from normal tissues served as the reference for CNV estimation. The cutoff was set to 0.1 and the other parameters were determined using the default settings of InferCNV.

#### Gene set activity analysis

Pathway analyses were performed on the 70 metabolic pathways as described.^72^ To assignan estimated value of pathway activity to individual cells, we applied AUCell (version 1.14.0) using the default settings.^73^ In addition, gene set variation analysis (GSVA; version 1.40.1) was employed to estimate pathway activity for The Cancer Genome Altas (TCGA) dataset.^74^ Significantly perturbed pathways were identified using the Limma R package (version 3.48.3) with a Benjamini–Hochberg-corrected *p* value ≤0.01.^64^

#### Public data acquisition

Independent scRNA-seq datasets and bulk RNA-seq data were acquired from publicly available sources. The scRNA-seq data for normal breast tissues were obtained from the GEO Database under accession numbers GSM7500359, GSM7500360, GSM7500361 and GSM7500391.^34^ scRNA-seq data were downloaded from the GSE176078 dataset.^4^ For validating metabolic findings in BC tissues, patient IDs and characteristics were included in Table S1. For exploring the role of nucleotide metabolism in the tumor microenvironment of various subtypes of BC, patient IDs and characteristics were included in Table S2. Bulk RNA-seq data were sourced from The Cancer Genome Atlas Program.

#### Plasma collection

All blood samples were collected using vacutainer EDTA Tubes (BD, USA) to facilitate plasma separation after an overnight fasting period of at least 8 h. For enrolled BC patients, 4 mL of peripheral blood was collected before the surgery, while for the NC control group, blood was collected in the morning during a routine health examination.

The whole blood samples were subjected to a two-step centrifugation process. They were first centrifuged at 1600g for 10 min at 4°C, and then the resulting supernatant (plasma) was further centrifuged at 16,000g for 10 min at 4°C. Aliquots of the supernatant were carefully transferred into cryovials and promptly stored at −80°C for subsequent analysis.

#### Sample preparation for metabolomics

A methanol-based extraction method was employed for sample preparation as described in previous studies,^75^^,^^76^^,^^77^ owing to its ability to achieve high metabolite coverage. For untargeted metabolomics profiling, metabolites were extracted by combining 400 μL of methanol, pre-cooled overnight at −20°C, with 100 μL of plasma (80% methanol). The resulting mixture was vigorously vortexed and shaken for 20 min, followed by centrifugation at 12,000 rpm for 20 min at 4°C to precipitate the proteins. All steps were performed at 4°C. Subsequently, 350 μL of the supernatant was dried under vacuum and stored at −80°C until further analysis. Additionally, QC samples were prepared by pooling small aliquots from each individual sample to assess the stability of the data throughout the analytical study.

For targeted metabolomics, metabolites were extracted from 50 μL of plasma using 200 μL of pre-cooled (−20°C) methanol, containing ^13^C5-inosine (5 ng/mL), ^13^C5-uridine (250 ng/mL), d8-Phe (500 ng/mL), and ^15^N-Thr (500 ng/mL) as internal standards. Following vortexing and shaking for 20 min, the mixture was centrifuged at 12,000 rpm for 20 min at 4°C. Then, 150 μL of the resulting supernatant was evaporated under vacuum and stored at −80°C for subsequent analysis. For the absolute quantification of selected metabolites, 13 calibrators for each analyte, used to construct calibration curves, were freshly processed on the day of the experiment. The concentration of the calibrators and the quantitative range of the assay were determined based on the expected concentrations obtained from preliminary experiments. Furthermore, QC samples at four different concentration levels (low, low medium, high medium, high) relative to the calibration range were freshly prepared using known quantities of analyte standards. Both calibrators and QC samples were prepared from separate stock solutions and subjected to the same processing protocol as the subject samples. Each analytical run included calibration curves and QC samples.

#### Untargeted metabolomics profiling

For untargeted metabolomics analysis, an LC-MS system comprised an Ultimate 3000 ultrahigh-performance LC (UHPLC) system coupled to a Q-Exactive mass spectrometer (Thermo Fisher Scientific). Metabolite extracts were reconstituted with 100 μL of a water/acetonitrile mixture (1:1, v/v). Following centrifugation at 12,000 rpm for 20 min, 5 μL of the supernatant was injected onto an XBridge BEH Amide column (100 × 2.1 mm, 2.5 μm; Waters) for metabolite separation at 30°C. A gradient elution method was employed using two mobile phases: (A) 5% acetonitrile in water with 5 mM ammonium acetate and (B) 100% acetonitrile. The linear gradient program was as follows: 0 min, 95% B; 2 min, 95% B; 15 min, 50% B; 18 min, 50% B; 19 min, 95% B; 23 min, 95% B. The flow rate was set to 0.35 mL/min.

MS/MS analysis was conducted in DDA mode using a positive/negative ion switching method on the Q-Exactive MS (Thermo Scientific). Except for polarity, the settings for Full MS and dd-MS/MS were identical in positive and negative ion modes. A survey scan (MS1 full-scan) was performed at a resolution of 35,000 in the m/z range of 60–800, followed by top 8 MS/MS scans in HCD mode at a resolution of 17,500. The automatic gain control (AGC) target for the MS1 and MS/MS scans was set to 5 × 10^6^ (with a maximum injection time of 100 ms) and 2e5 (with a maximum injection time of 64 ms), respectively. Dynamic exclusion was set to 8 s, and stepped normalized collision energy (NCE) was set to 15, 30, and 45. The parameters of the HESI ion source in ESI mode were configured as follows: capillary temperature, 320°C; auxiliary heater temperature, 300°C; sheath gas flow rate, 40 arb; auxiliary gas flow rate, 10 arb; sweep gas flow rate, 2 arb; S-lens RF level, 55. A QC sample was analyzed before and after the sequence and approximately every 10 sample runs in the sequence to assess the stability of the LC-MS instrument.

#### Targeted metabolomics quantification

For targeted metabolomics analysis, a Jasper HPLC System (AB SCIEX) coupled with a 4500MD QTRAP triple quadrupole mass spectrometer (AB SCIEX) was employed. The LC method was further optimized to specifically target metabolites, ensuring optimal chromatographic peak shape and quantitative accuracy. Metabolite extracts were reconstituted and diluted four times using 200 μL of a water/acetonitrile mixture (1:3, v/v), and 2 μL of the resulting supernatant was injected onto an XBridge BEH Amide column (100 × 2.1 mm, 2.5 μm; Waters) at 30°C. The mobile phases used were as follows: mobile phase A contained 5% acetonitrile with 1 mM ammonium acetate and 0.1% formic acid in water, while mobile phase B consisted of 100% acetonitrile. The linear gradient program was as follows: 0 min, 95% B; 2 min, 95% B; 9 min, 40% B; 10 min, 40% B; 11 min, 40% B; 12 min, 95% B; 19 min, 95% B. The flow rate was set at 0.35 mL/min. Targeted quantification of selected metabolites was performed in MRM mode. After optimizing the source gas parameters, DP and CE of the compounds, two optimal transitions were selected for each of the 4 targeted metabolites and their corresponding SIL-IS. A total of 16 transitions were detected in positive ion mode during a 19-min LC-MRM-MS run. The optimized source gas parameters were as follows: temperature, 500°C; ion spray voltage, 5.5 kV; gas1, 55; gas2, 60; curtain gas, 35. The MRM parameters are listed in Table S5.

#### Raw data processing

The raw data obtained from untargeted metabolomics analysis were converted into the standardized file format of Reifycs Inc. using the Reifycs ABF converter (http://www.reifycs.com/AbfConverter/index.html). Subsequently, the data were processed using MS-DIAL software (version 4.70), which incorporates spectral deconvolution, metabolite identification, and peak alignment between samples as outlined in the software tutorial.^61^ Metabolite identification based on MS/MS spectra was performed in MS-DIAL by comparing the acquired MS/MS spectra with the software’s internal in silico MS/MS spectra database (MSMS-Pos/Neg-MassBank.msp; MSP format version July 20, 2021). Mass accuracy tolerances of 0.01 Da and 0.05 Da were set for MS1 and MS2 data collection and identification, respectively. For peak alignment with the QC file specified in the reference file, retention time and MS1 tolerances were set to 0.2 min and 0.01 Da, respectively. In cases of missing values, the option to replace them with 1/10 of the minimum peak height across all samples was selected when exporting the raw area data matrices. Subsequently, the alignment results were processed using statTarget analysis, employing the QC-robust LOESS signal correction methods along with the QC-based random forest signal correction algorithm (QC-RFSC).^35^ Default settings and parameters were used.

The acquired targeted MRM data was processed using MultiQuant MD 3.0.3 software (AB SCIEX) for automated LC-MS peak integration. Manual review of all chromatographic peaks was performed to assess integration quality, comparing them to analyte standards and SIL-IS of each metabolite. The ratios of peak areas between the analyte product ion response and the corresponding SIL-IS product ion response were calculated and utilized for calibration and quantification purposes. SIL-IS-based calibration curves were generated through weighted least squares linear regression of these ratios and the expected concentrations of the analytes. A weighting factor of 1/x^2^ was employed, following the recommendation for all LC-MS bioanalytical assays in previous studies.^78^ The back-calculated concentrations were required to be within ±15% (±20% for lower limits of quantification, LLOQ) of the nominal values in at least 75% of the calibrators (Tables S9 and S10). The concentrations of analytes in the QC samples and subject samples were determined using the resulting calibration curves, and a two-dimensional matrix was exported as an Excel file.

#### Support vector machine and feature selection

SVM is a type of generalized linear classifier employed for supervised learning, wherein it determines a decision boundary known as the maximum margin hyperplane based on the training samples. SVM utilizes a linear kernel to train the model. In this study, we constructed an SVM model (liblinear 220) for the classification of BC and NC samples.^67^ Prior to training, the data underwent normalization using L2 normalization, which is achieved through the following equation, where fi represents the *i*th dimension of the data for each sample, and M denotes the dimension of the data.

$$f_{i}=\frac{f_{i}}{\sqrt{\sum_{m=1}^{M}f_{m}^{2}}}$$

The SVM algorithm determined the "maximum margin" or hyperplane capable of separating a sample set using the following equation, where x_i_ represents the data and y_i_ denotes the label for *i*th sample. $\min_{w,b}\frac{{\left\|w\right\|}^{2}}{2},s.t.\;\left(w^{T}x_{i}+b\right)y_{i}\geq 1$

As illustrated in the above equation, the inferred coefficient *w* could be utilized as a weight to assess the significance of each feature, enabling the selection of features that contribute more prominently to the model’s classification. Consequently, predictive models for feature selection were developed by progressively increasing and selecting the top-ranking features based on their attribute weights. This iterative process continued until the top 100 features were included as the 100th model. The hyperparameter was set to 4. To evaluate the performance, 500 iterations of 4-fold cross-validation were conducted, and the average accuracy of each model (*N* = 100) in feature selection was calculated.

### Quantification and statistical analysis

PLS-DA was performed using a general linear model on the MetaboAnalyst 6.0 online service (https://www.metaboanalyst.ca/).^62^ In the normalization procedure, we select.

“Log transformation (base 10)” for data transformation, and select “none” for sample normalization and data scaling. Coss validation and permutation test were used for performance evaluation of classification models. 5-fold cross validation was applied and Q^2^ was selected as the performance measure. The optimal number of components needed to build the model was marked by a red star. In the process of permutation test, the within-group sum of squares (B/W-ratio) was selected as the test statistic, and the permutation number was set to 1000. A *p* value < 0.05 was considered statistically significant.

Metabolite set enrichment analysis was performed using the MetaboAnalyst 6.0 online service (https://www.metaboanalyst.ca/). A concentration table containing all detected metabolites was uploaded to the module of quantitative enrichment analysis. The dataset was normalized by median and transformed by using a standard log transformation (base 10). The KEGG library was selected as the metabolite set library and only the metabolite sets containing at least 2 entries were used. The *p* values presented were FDR adjusted based on Benjamini–Hochberg procedure.

MATLAB R2022b, Graphpad Prism v 9.0.0 and SPSS R26.0.0 software packages were used for statistical analysis. For the univariate analysis, *t*-test was applied to calculate the statistical significance, and the significance levels were indicated as follows: ∗*p* < 0.05, ∗∗*p* < 0.01, ∗∗∗*p* < 0.001, ∗∗∗∗*p* < 0.0001. False Discovery Rate (FDR) correction was applied when multiple comparisons were conducted. The adjusted *p* values were calculated using the Benjamini-Hochberg method, with significance levels adjusted accordingly. And FDR-adjusted *p* value cutoff was set to 0.05. Pearson correlation analysis was performed to assess the associations between metabolic pathway activity and gene expressions. For the targeted assay data, t-SNE analysis was performed using the t-SNE function in MATLAB. The statistical tools, methods, and thresholds employed for each analysis of scRNA-seq data are explicitly described in the Results section and in the Supplementary Materials.

## Supplemental information

Document S1. Figures S1–S10, Tables S3, S5–S9, and S11

Table S1. Characteristics of five patients with breast cancer used for single cell sequencing and six samples from GSE176078 for validation, related to Figure 2, Figure S3 and STAR Methods

Table S2. Table S2. Characteristics of nineteen breast cancer samples of the open-source scRNA-seq dataset, related to Figure 2, Figure S4-S5 and STAR Methods

Table S4. Sample source and characteristics of the subjects in each cohort, related to Figure S1, Figure 5 and STAR Methods

Table S10. Table S10. Detailed information of the four standard curves, related to Figure S10 and STAR Methods
