# Supplementary material for: Multi-omic analysis identifies metabolic biomarkers for the early detection of breast cancer and therapeutic response prediction
Source: iScience. 2024 Aug 5;27(9):110682. doi: 10.1016/j.isci.2024.110682 (PMC11381768; doi:10.1016/j.isci.2024.110682)
Supplement: Supplementary file 1 — Document S1. Figures S1–S10, Tables S3, S5–S9, and S11 [file mmc1.pdf]

iScience, Volume *27*

Supplemental information

Multi-omic analysis identifies metabolic biomarkers for the early detection of breast cancer and therapeutic response prediction

Huajie Song, Xiaowei Tang, Miao Liu, Guangxi Wang, Yuyao Yuan, Ruifang Pang, Chenyi Wang, Juntuo Zhou, Yang Yang, Mengmeng Zhang, Yan Jin, Kewei Jiang, Shu Wang, and Yuxin Yin

Supplemental figures and legends

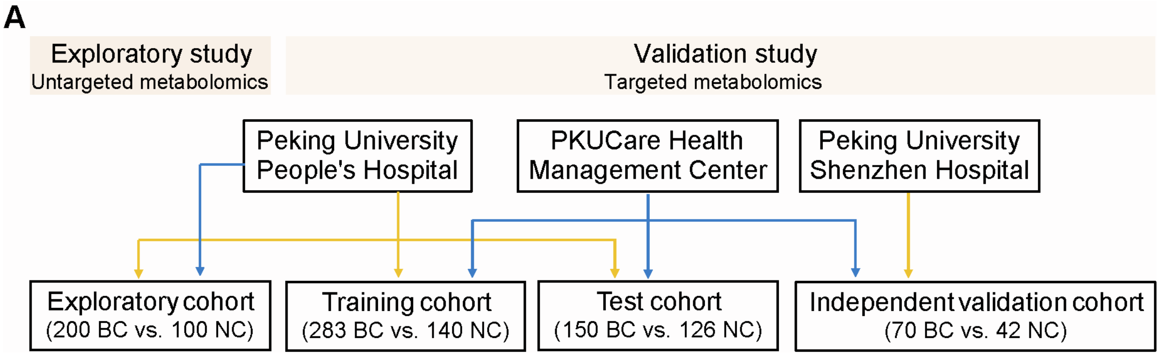

Figure S1. Participants of BC patients and NC, related to Figure 1 and Table S4.
(A) Flow chart of BC patients and NC enrolled in the SVM-based metabolic BC detection approach (yellow lines for BC, blue lines for NC).

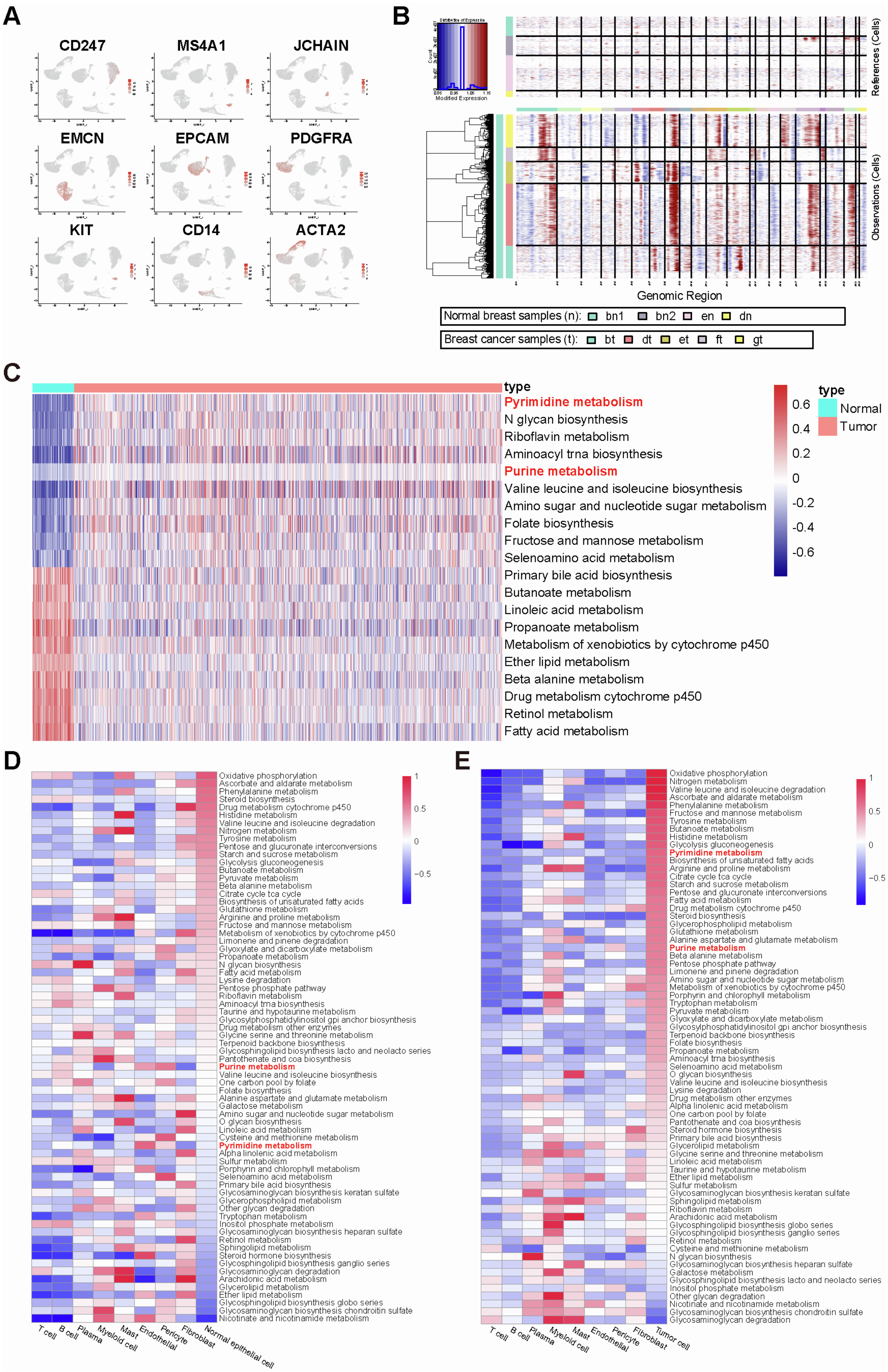
A
CD247
MS4A1
JCHAIN
EMCN
EPCAM
PDGFRA
KIT
CD14
ACTA2
B
Modified Expression
References (Cells)
Observations (Cells)
Genomic Region
Normal breast samples (n): bn1 bn2 en dn
Breast cancer samples (t): bt dt et ft gt
C
type
Pyrimidine metabolism
N glycan biosynthesis
Riboflavin metabolism
Aminoacyl trna biosynthesis
Purine metabolism
Valine leucine and isoleucine biosynthesis
Amino sugar and nucleotide sugar metabolism
Folate biosynthesis
Fructose and mannose metabolism
Selenoamino acid metabolism
Primary bile acid biosynthesis
Butanoate metabolism
Linoleic acid metabolism
Propanoate metabolism
Metabolism of xenobiotics by cytochrome p450
Ether lipid metabolism
Beta alanine metabolism
Drug metabolism cytochrome p450
Retinol metabolism
Fatty acid metabolism
0.6
0.4
0.2
0
-0.2
-0.4
-0.6
type
Normal
Tumor
D
Oxidative phosphorylation
Ascorbate and aldarate metabolism
Phenylalanine metabolism
Steroid biosynthesis
Drug metabolism cytochrome p450
Histidine metabolism
Valine leucine and isoleucine degradation
Nitrogen metabolism
Tyrosine metabolism
Pentose and glucuronate interconversions
Starch and sucrose metabolism
Glycolysis gluconeogenesis
Butanoate metabolism
Pyruvate metabolism
Beta alanine metabolism
Citrate cycle tca cycle
Biosynthesis of unsaturated fatty acids
Glutathione metabolism
Arginine and proline metabolism
Fructose and mannose metabolism
Metabolism of xenobiotics by cytochrome p450
Limonene and pinene degradation
Glyoxylate and dicarboxylate metabolism
Propanoate metabolism
N glycan biosynthesis
Fatty acid metabolism
Lysine degradation
Pentose phosphate pathway
Riboflavin metabolism
Aminoacyl trna biosynthesis
Taurine and hypotaurine metabolism
Glycosylphosphatidylinositol gpi anchor biosynthesis
Drug metabolism other enzymes
Glycine serine and threonine metabolism
Terpenoid backbone biosynthesis
Glycosphingolipid biosynthesis lacto and neolacto series
Pantothenate and coa biosynthesis
Purine metabolism
Valine leucine and isoleucine biosynthesis
One carbon pool by folate
Folate biosynthesis
Alanine aspartate and glutamate metabolism
Galactose metabolism
Amino sugar and nucleotide sugar metabolism
O glycan biosynthesis
Linoleic acid metabolism
Cysteine and methionine metabolism
Pyrimidine metabolism
Alpha linolenic acid metabolism
Sulfur metabolism
Porphyrin and chlorophyll metabolism
Selenoamino acid metabolism
Primary bile acid biosynthesis
Glycosaminoglycan biosynthesis keratan sulfate
Glycerophospholipid metabolism
Other glycan degradation
Tryptophan metabolism
Inositol phosphate metabolism
Glycosaminoglycan biosynthesis heparan sulfate
Retinol metabolism
Sphingolipid metabolism
Steroid hormone biosynthesis
Glycosphingolipid biosynthesis ganglio series
Glycosaminoglycan degradation
Arachidonic acid metabolism
Glycerolipid metabolism
Ether lipid metabolism
Glycosphingolipid biosynthesis globo series
Glycosaminoglycan biosynthesis chondroitin sulfate
Nicotinate and nicotinamide metabolism
1
0.5
0
−0.5
T cell
B cell
Plasma
Myeloid cell
Mast
Endothelial
Pericyte
Fibroblast
Normal epithelial cell
E
Oxidative phosphorylation
Nitrogen metabolism
Valine leucine and isoleucine degradation
Ascorbate and aldarate metabolism
Phenylalanine metabolism
Fructose and mannose metabolism
Tyrosine metabolism
Butanoate metabolism
Histidine metabolism
Glycolysis gluconeogenesis
Pyrimidine metabolism
Biosynthesis of unsaturated fatty acids
Arginine and proline metabolism
Citrate cycle tca cycle
Starch and sucrose metabolism
Pentose and glucuronate interconversions
Fatty acid metabolism
Drug metabolism cytochrome p450
Steroid biosynthesis
Glycerophospholipid metabolism
Glutathione metabolism
Alanine aspartate and glutamate metabolism
Purine metabolism
Beta alanine metabolism
Pentose phosphate pathway
Limonene and pinene degradation
Amino sugar and nucleotide sugar metabolism
Metabolism of xenobiotics by cytochrome p450
Porphyrin and chlorophyll metabolism
Tryptophan metabolism
Pyruvate metabolism
Glyoxylate and dicarboxylate metabolism
Glycosylphosphatidylinositol gpi anchor biosynthesis
Terpenoid backbone biosynthesis
Folate biosynthesis
Propanoate metabolism
Aminoacyl trna biosynthesis
Selenoamino acid metabolism
O glycan biosynthesis
Valine leucine and isoleucine biosynthesis
Lysine degradation
Drug metabolism other enzymes
Alpha linolenic acid metabolism
One carbon pool by folate
Pantothenate and coa biosynthesis
Steroid hormone biosynthesis
Primary bile acid biosynthesis
Glycerolipid metabolism
Glycine serine and threonine metabolism
Linoleic acid metabolism
Taurine and hypotaurine metabolism
Ether lipid metabolism
Sulfur metabolism
Glycosaminoglycan biosynthesis keratan sulfate
Sphingolipid metabolism
Riboflavin metabolism
Arachidonic acid metabolism
Glycosphingolipid biosynthesis globo series
Glycosphingolipid biosynthesis ganglio series
Retinol metabolism
Cysteine and methionine metabolism
N glycan biosynthesis
Glycosaminoglycan biosynthesis heparan sulfate
Galactose metabolism
Glycosphingolipid biosynthesis lacto and neolacto series
Inositol phosphate metabolism
Other glycan degradation
Nicotinate and nicotinamide metabolism
Glycosaminoglycan biosynthesis chondroitin sulfate
Glycosaminoglycan degradation
1
0.5
0
−0.5
T cell
B cell
Plasma
Myeloid cell
Mast
Endothelial
Pericyte
Fibroblast
Tumor cell

Figure S2. Expression profiles of metabolic pathways in scRNA-seq analyses and enrichment scores in TCGA dataset, related to Figure 2 and Tables S1-3.

(A) Identification of cell clusters using canonical markers for different cell types, as shown in the UMAP plot.

(B) Heatmap displaying normalized large-scale CNVs in indicated cell types from BC patients. Reference cells are normal epithelial cells from 4 NC (upper panel). Large-scale CNV are observed in epithelial cells in BC samples (lower panel). Red indicates high CNV levels, while blue indicates low CNV levels.

(C) The heatmap showing the enrichment scores of the metabolic pathways in the TCGA dataset. Pathways are labeled with name and sorted by t-values. The top 10 enriched KEGG terms are presented.

(D-E) Metabolic pathway expression profiles in normal breast tissues (left panel) and BC (right panel). For each pathway, the fold change in epithelial cells (normal tissues, n = 4) or tumor cells (BC, n=5) was calculated relative to other cell types and corrected for sample of origin. Pathways were ordered by log-fold change in normal epithelial and tumor cells, respectively.

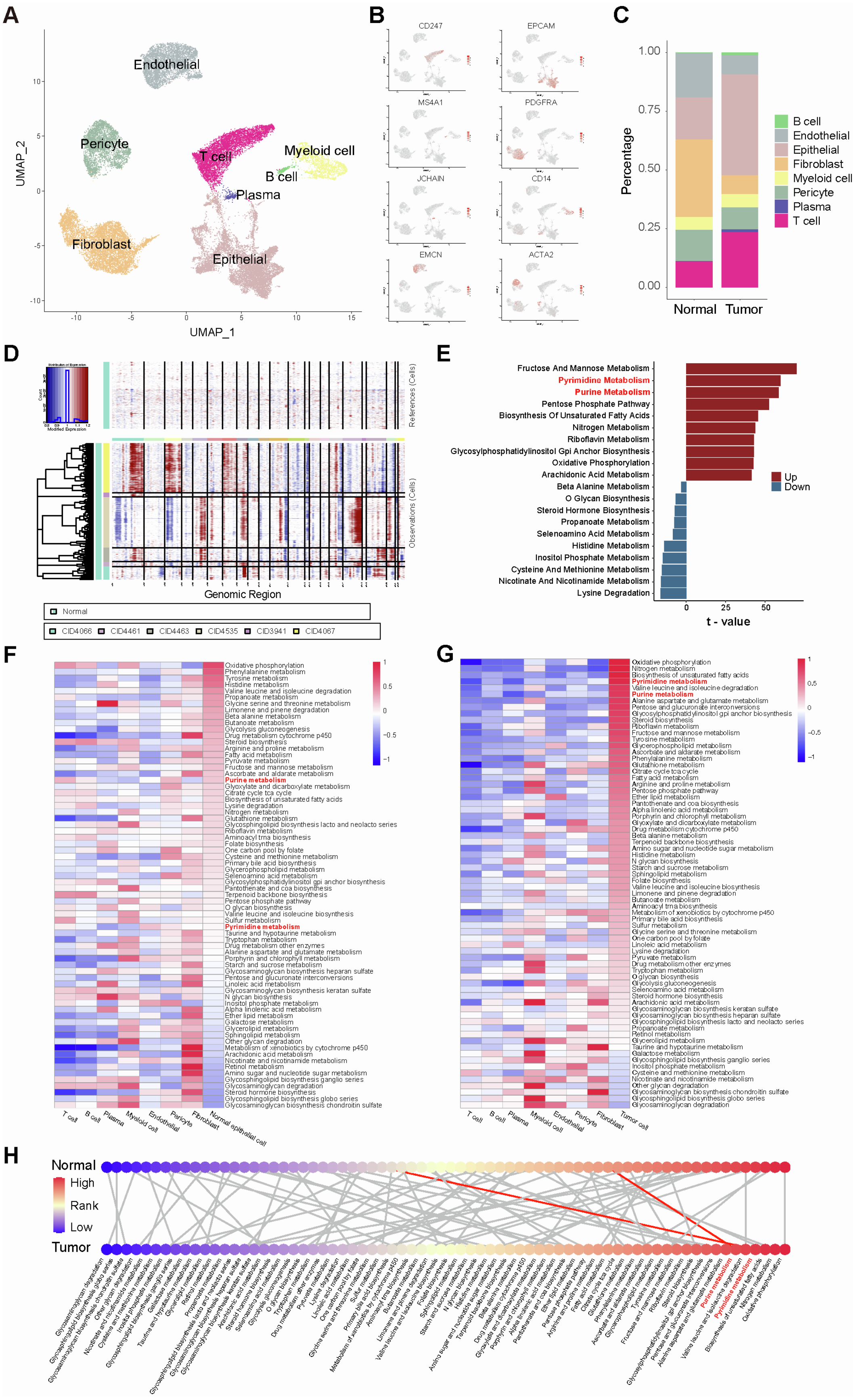
A
Endothelial
Pericyte
T cell
Myeloid cell
B cell
Plasma
Fibroblast
Epithelial
UMAP_1
UMAP_2
B
CD247
EPCAM
MS4A1
PDGFRA
JCHAIN
CD14
EMCN
ACTA2
C
Percentage
Normal
Tumor
B cell
Endothelial
Epithelial
Fibroblast
Myeloid cell
Pericyte
Plasma
T cell
D
References (Cells)
Observations (Cells)
Genomic Region
Normal
CID4066
CID4461
CID4463
CID4535
CID3941
CID4067
E
Fructose And Mannose Metabolism
Pyrimidine Metabolism
Purine Metabolism
Pentose Phosphate Pathway
Biosynthesis Of Unsaturated Fatty Acids
Nitrogen Metabolism
Riboflavin Metabolism
Glycosylphosphatidylinositol Gpi Anchor Biosynthesis
Oxidative Phosphorylation
Arachidonic Acid Metabolism
Beta Alanine Metabolism
O Glycan Biosynthesis
Steroid Hormone Biosynthesis
Propanoate Metabolism
Selenoamino Acid Metabolism
Histidine Metabolism
Inositol Phosphate Metabolism
Cysteine And Methionine Metabolism
Nicotinate And Nicotinamide Metabolism
Lysine Degradation
Up
Down
t - value
F
T cell
B cell
Plasma
Myeloid cell
Endothelial
Pericyte
Fibroblast
Normal epithelial cell
G
T cell
B cell
Plasma
Myeloid cell
Endothelial
Pericyte
Fibroblast
Tumor cell
H
Normal
High
Rank
Low
Tumor

Figure S3. Validation of scRNA-seq metabolism findings in six BC samples from an external scRNA-seq dataset, related to Figure 2 and Tables S1.

(A) Identification of cell populations in human breast tissues. The UMAP visualization depicts 15,116 cells from 6 breast tumor samples and 19,496 cells from 4 adjacent nonmalignant breast tissues, revealing eight major cell clusters labeled by cell type. Each dot represents an individual cell and is color coded accordingly.

(B) Canonical markers for the different cell types were used to identify cell clusters, as illustrated in the UMAP plot.

(C) Average proportion of different cell types in normal breast samples (n = 4) and BC tissues (n = 6).

(D) Heatmap of normalized large-scale CNVs. Normalized large-scale copy number variations (CNVs) in specific cell types from BC patients are presented. Reference cells are normal epithelial cells from 4 NC samples (upper panel), whereas large-scale CNVs are observed in epithelial cells in BC cancer samples (lower panel). High CNV levels are indicated in red, while low levels are indicated in blue.

(E) AUCell analysis was performed on up-regulated and down-regulated pathways. Metabolism-related gene sets are labeled by name and sorted by t-values. The top 10 upregulated and downregulated metabolic pathways are displayed.

(F-G) Metabolic pathway expression profiles in normal breast tissues (left panel) and BC (right panel). For each pathway, the fold change in epithelial cells (normal tissues, n = 4) or tumor cells (BC, n=6) was calculated relative to other cell types and corrected for sample of origin. Pathways were ordered by log-fold change in normal epithelial and tumor cells, respectively.

(H) Expression changes in metabolic pathways in tumor cells are depicted. These pathways are ordered by log-fold change in normal epithelial cells (upper panel) and tumor cells (lower panel), respectively. Solid lines connect the same pathway in normal and BC tissues, with red lines highlighting nucleotide metabolism. Each node represents a single pathway, and node color reflects its expression levels in normal epithelial cells or tumor cells compared to other cell types.

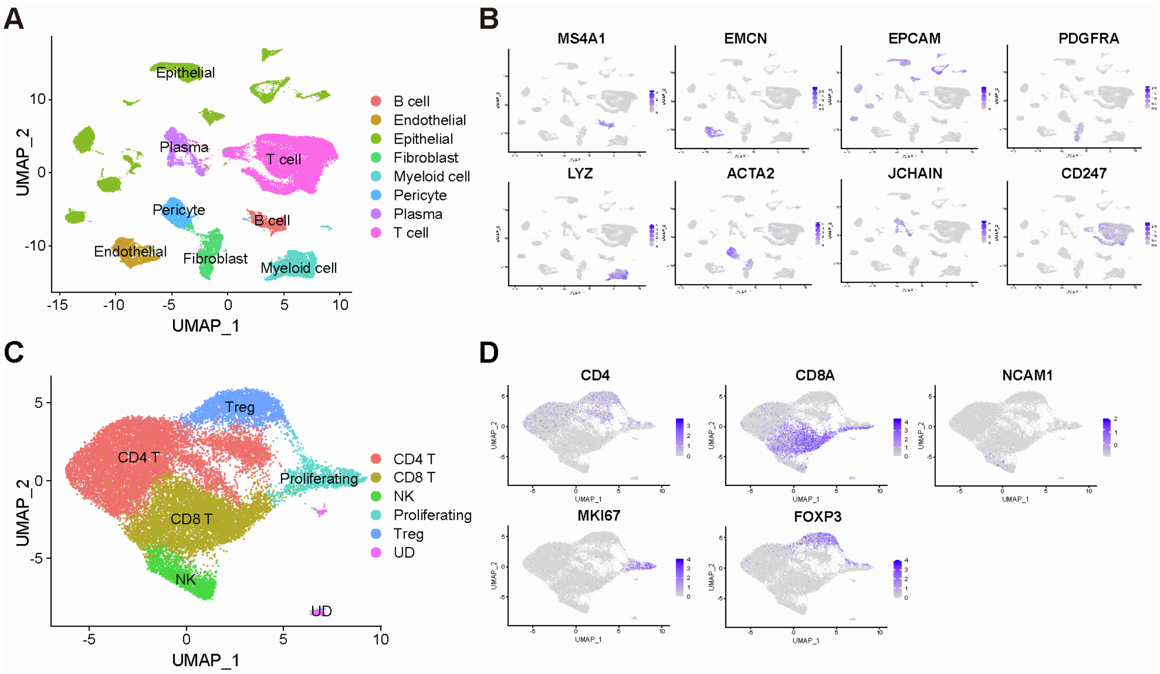

Figure S4. Details of clustering results of nineteen BC samples from an external scRNA-seq dataset, related to Figure 2 and Tables S2.

(A) The UMAP visualization of 64,738 single cells from nineteen primary breast tumors including 7 TNBCs, 8 HR+/HER2- and 4 HER2+, with eight major cell clusters identified and labeled.

(B) Canonical markers for different cell types were used to identify cell clusters, as shown in the UMAP plot.

(C) Shown is the UMAP visualization of various T-cell types, including 23,958 single cells with five major T-cell clusters identified and labeled.

(D) Canonical markers for various T-cell types were used to identify cell clusters, as shown in the UMAP plot.

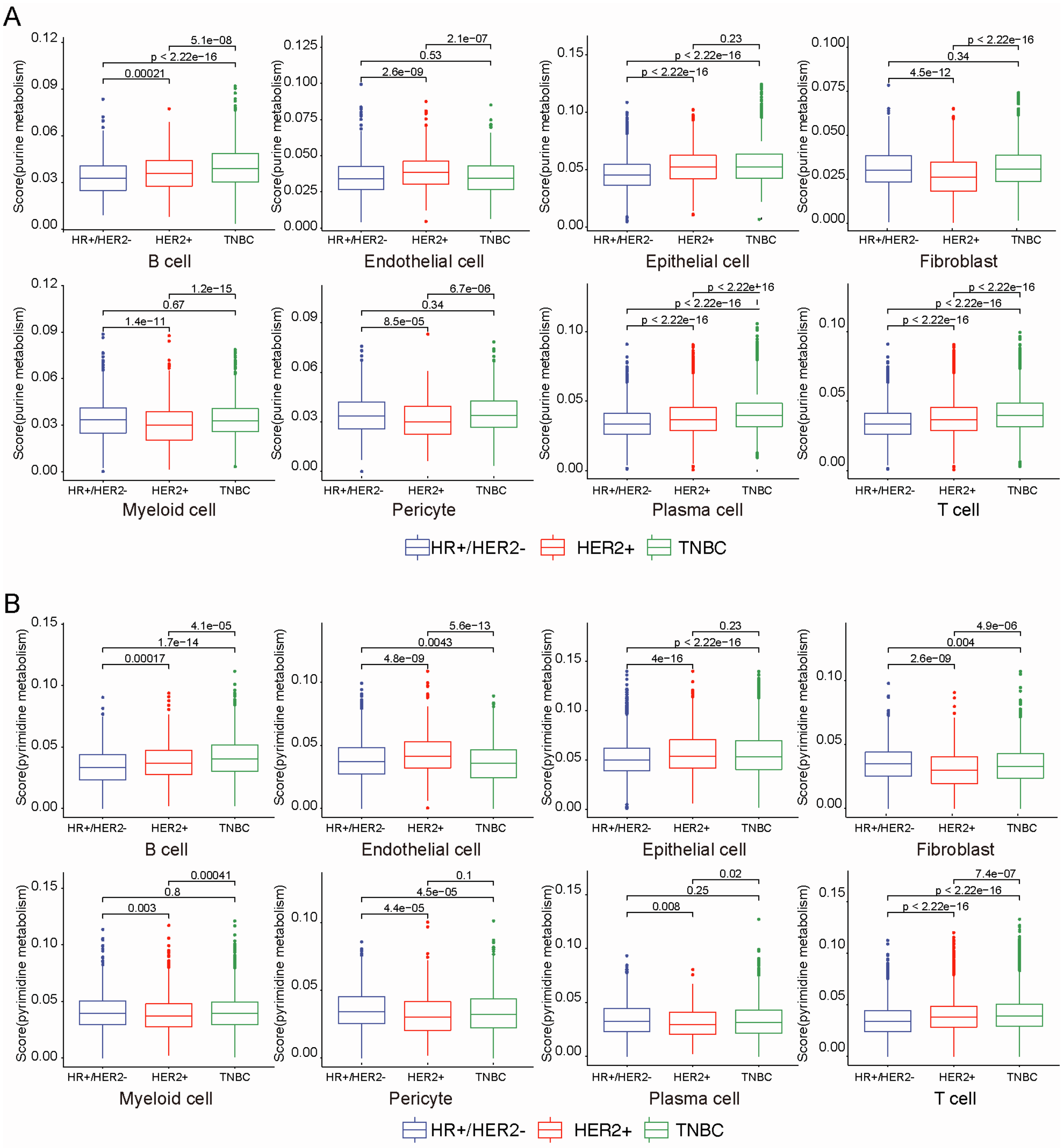

Figure S5. Expression of purine metabolism (A) and pyrimidine metabolism (B) in each cell type among three subtypes of nineteen BC patients from an external scRNA-seq dataset (*t*-test), related to Figure 2 and Tables S2.

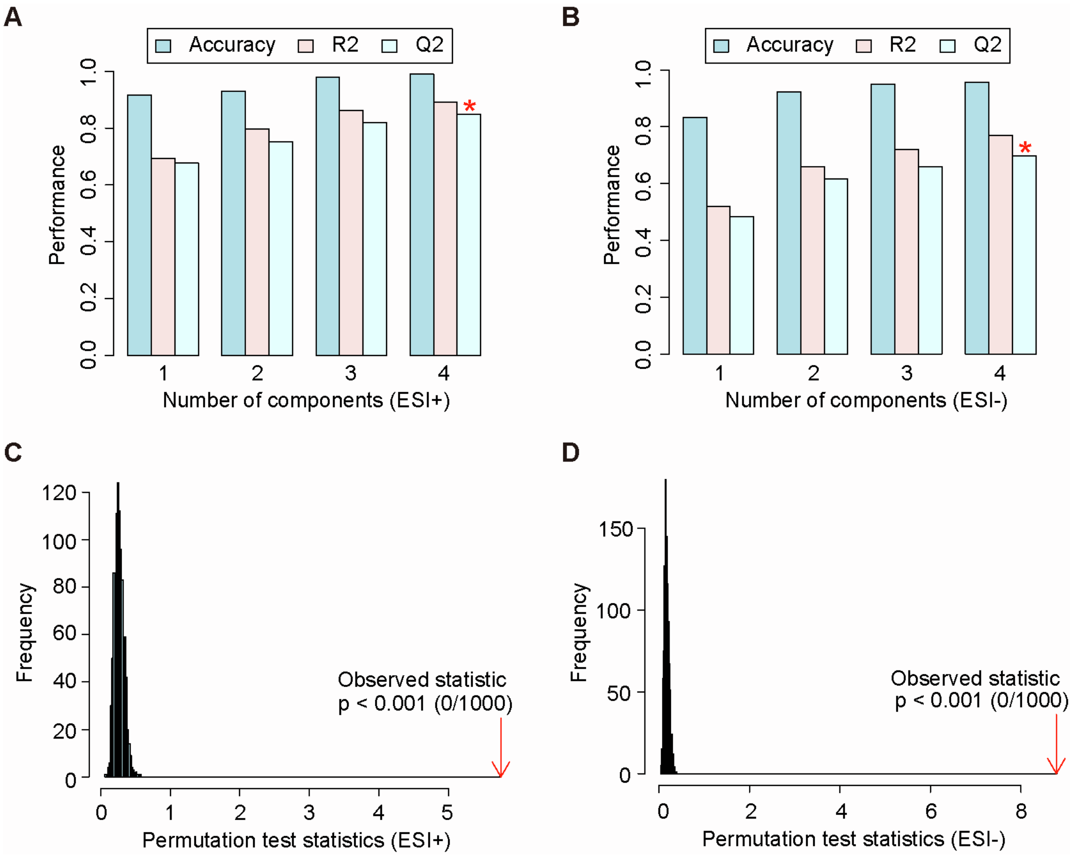

Figure S6. The performance evaluation of the PLS-DA models in untargeted metabolomic analysis, related to Figure 3.

(A and B) Cross validation results. The selected performance measure-Q2 shows that the four-component model is best for untargeted metabolomics data in ESI+ mode (A) and in ESI- mode (B) (indicated by a red star).

(C and D) Permutation test results. The histogram illustrates that the observed statistic, derived from the original data, falls outside the null distribution generated by permutations of the data in ESI+ mode (C) and in ESI- mode (D). The *p* value is significant (<0.001).

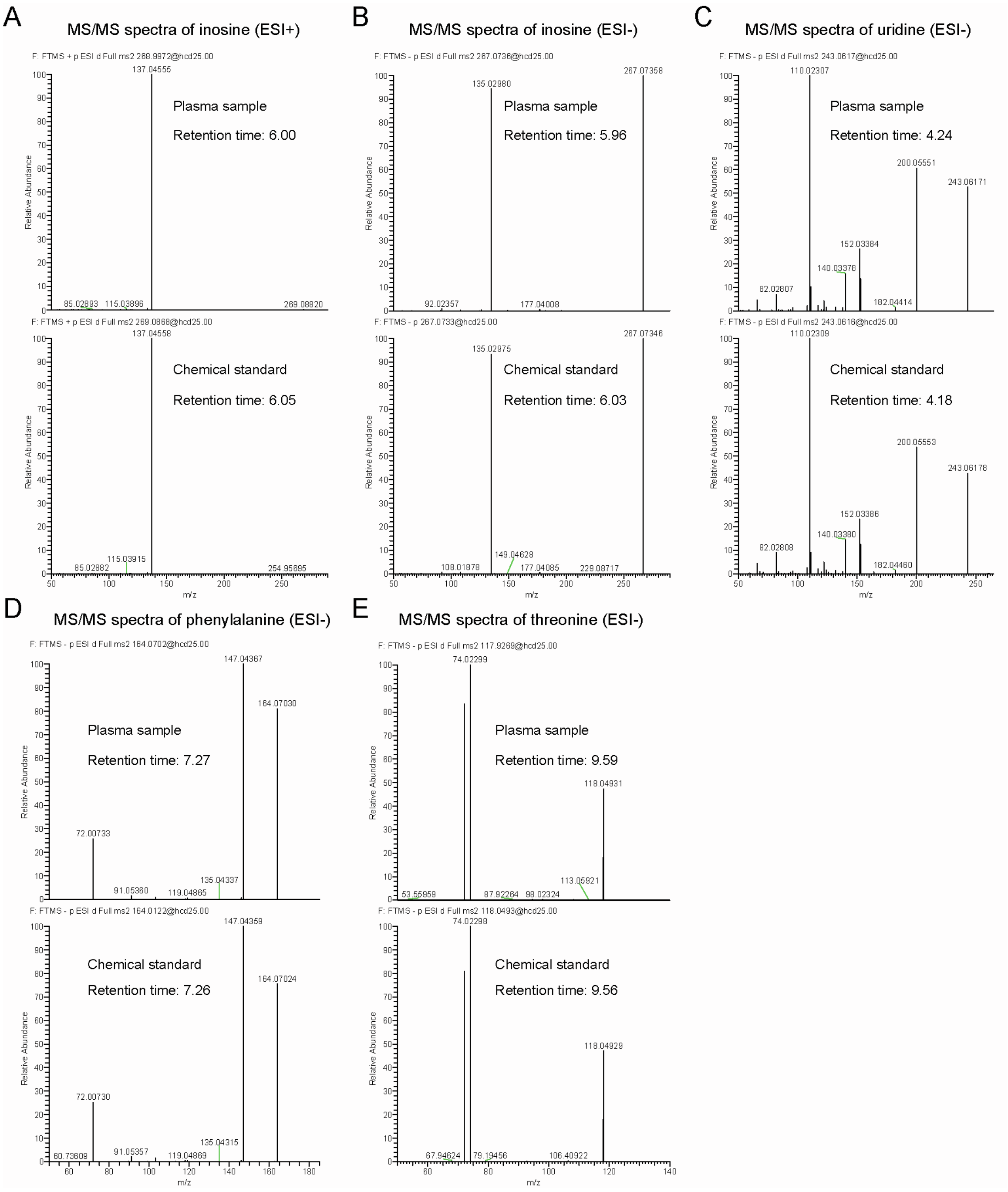

Figure S7. Validation of metabolite marker identities using chemical standards in DDA mode (untargeted metabolomics), related to Figure 4.

(A-E) Comparison of metabolites in plasma samples (upper panel) with chemical standards (lower panel). MS2 spectra and retention times of each metabolite are indicated on the respective panel for inosine in ESI+ mode (A) and in ESI- mode (B), as well as for uridine (C), phenylalanine (D), and threonine (E) in ESI- mode.

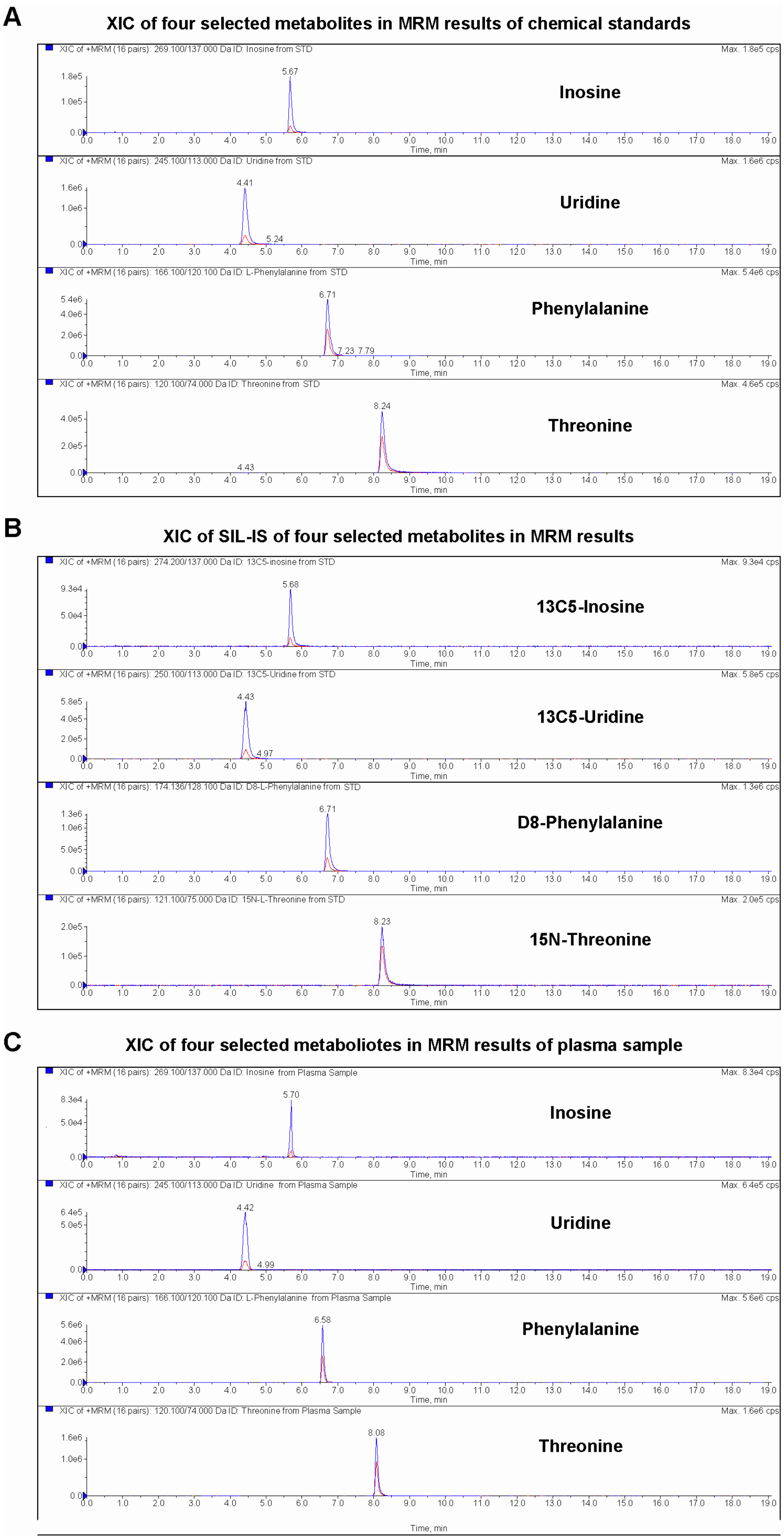
A
XIC of four selected metabolites in MRM results of chemical standards
Inosine
Uridine
Phenylalanine
Threonine
B
XIC of SIL-IS of four selected metabolites in MRM results
13C5-Inosine
13C5-Uridine
D8-Phenylalanine
15N-Threonine
C
XIC of four selected metaboliotes in MRM results of plasma sample
Inosine
Uridine
Phenylalanine
Threonine
Time, min

Figure S8. Validation of metabolite marker identities using chemical standards in MRM mode (targeted metabolomics), related to Figure 4 and Tables S7-8.

(A and C) Extracted ion chromatograms (XICs) for four selected metabolites in MRM results of chemical standards (A) and plasma samples (C).

(B) XICs of SIL-IS for four selected metabolites in MRM results.

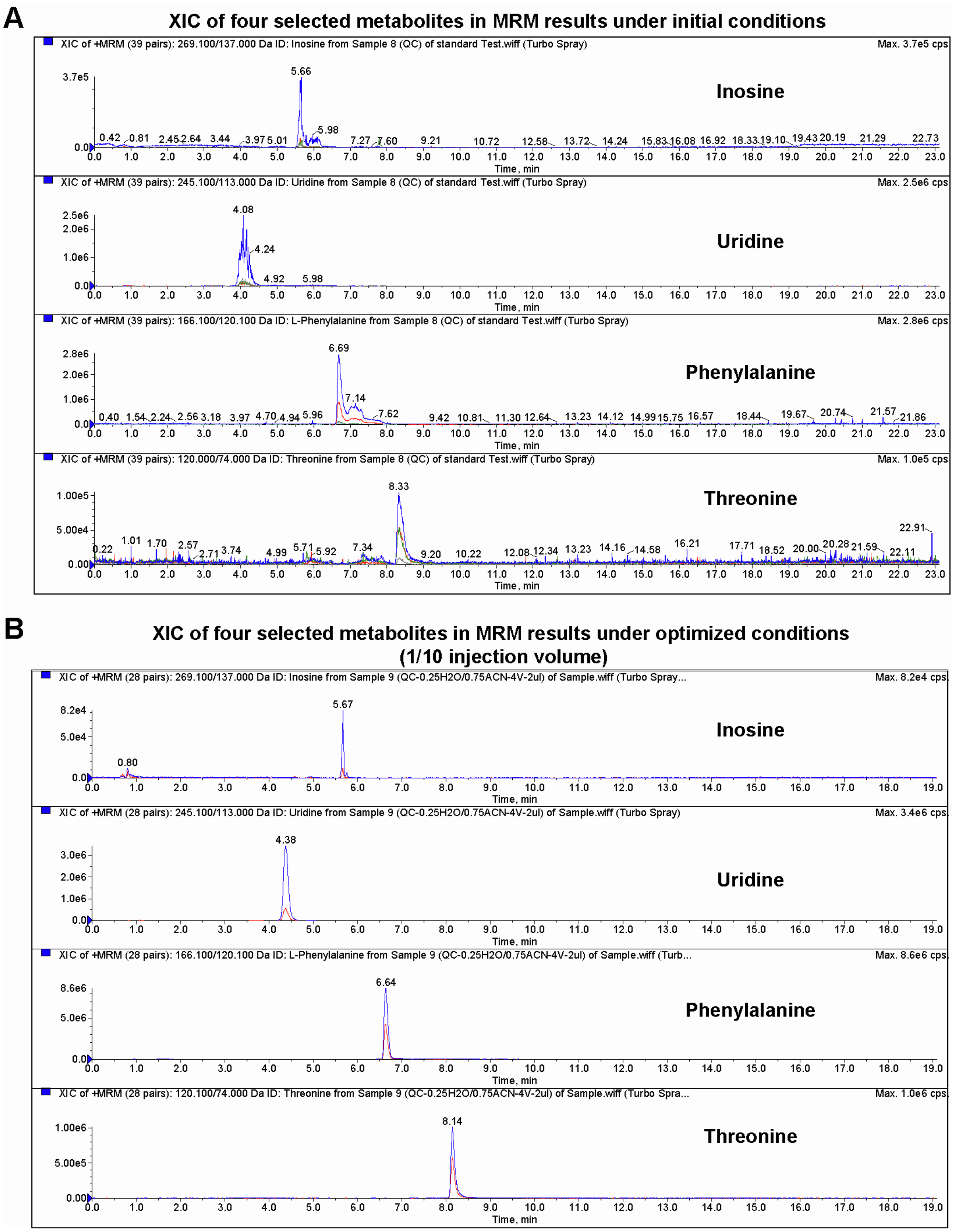

Figure S9. Comparative XICs of four selected metabolites in MRM results before and after method optimization, related to Figure 4.

(A) XICs of four selected metabolites under initial conditions.

(B) XICs of four selected metabolites under optimized conditions.

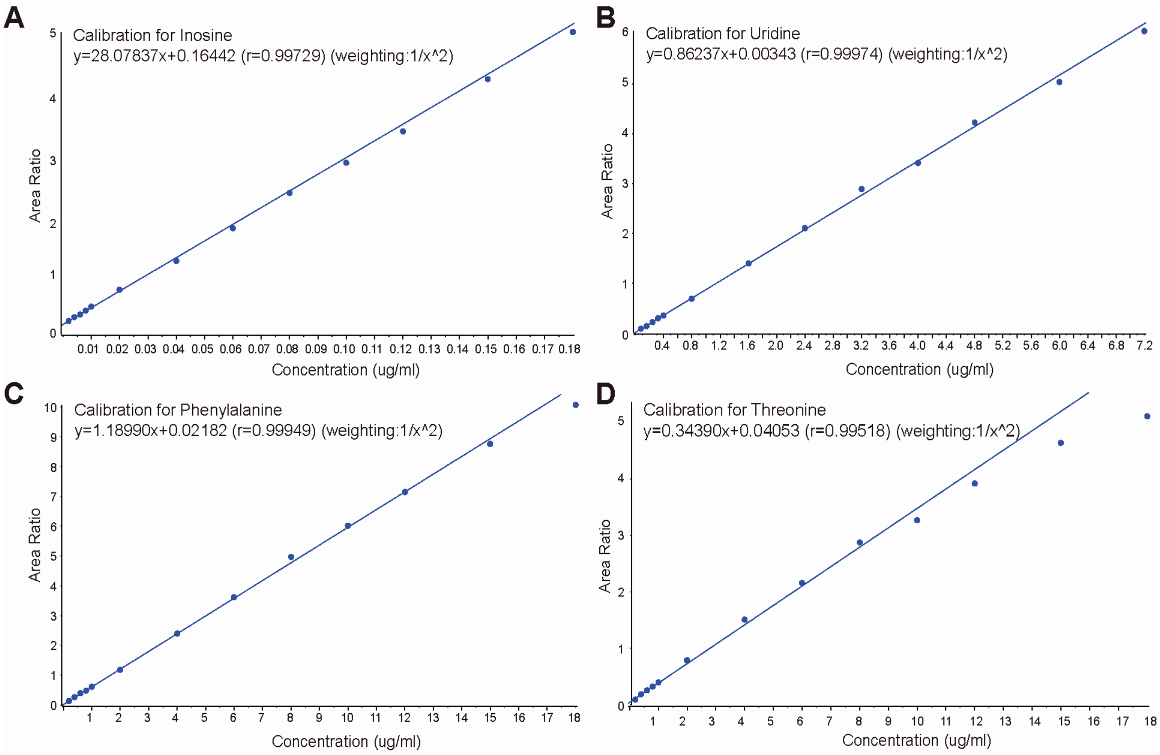

Figure S10. Standard curves for the four metabolite markers of the HILIC-MRM-MS targeted method, related to Figure 4 and Tables S9-10.

(A-D) Standard curves for inosine (A), uridine (B), phenylalanine (C), and threonine (D).

Supplemental tables

Table S3. Correlation analysis results of metabolic pathway activity in tumor cells and T-cell-related markers, related to Figure 2

Gene	cor_purine metabolism	*p* value_purine metabolism	cor_pyrimidine metabolism	*p* value_pyrimidine metabolism
TLR4	0.731665958	0.002936468	0.250743547	0.387224759
NFATC2	0.696077493	0.005690073	0.270298433	0.34997725
TLR10	0.679259137	0.007545278	0.295951349	0.304252329
ADORA3	0.674869389	0.008099009	0.444401376	0.111378501
ADORA2A	0.622306345	0.017473423	0.140036065	0.633020217
TCF7	0.56987308	0.033374668	0.405357221	0.15046178
FOXP3	0.564030818	0.035650072	0.012322318	0.966651539
CCR8	0.554379339	0.039657364	-0.001051262	0.997154207
ENTPD1	0.536469426	0.047956893	0.256137159	0.376748376
TLR2	0.532623373	0.049892418	0.555368399	0.03923215
IL2RA	0.532317914	0.050048536	-0.104675685	0.721746774
CD8A	0.511963258	0.061272273	0.155829771	0.594747359
LAG3	0.505096619	0.065436296	0.086172991	0.769588487
ADORA2B	0.489652302	0.075534178	-0.123433581	0.674192796
NFAT5	0.483894178	0.079566508	0.259446668	0.370396115
TNFRSF9	0.452303409	0.104413418	-0.008643111	0.976605741
TLR6	0.451367662	0.105222129	0.656643491	0.010742234
RTKN2	0.450744356	0.105763202	0.024243964	0.934435077
CD4	0.450447103	0.106021911	-0.129193523	0.659805453
PDCD1	0.448851999	0.107417619	-7.03E-05	0.999809573
NFATC1	0.443453183	0.112235095	0.044513437	0.879897728
NFATC3	0.429973685	0.12490288	0.310507194	0.279926016
GNLY	-0.427699989	0.127131011	-0.150635813	0.607232478
P2RY2	-0.41122027	0.144084595	-0.35456156	0.213549168
TOX	0.403682023	0.152317601	-0.112713556	0.701243844
NT5E	0.387097928	0.171507302	0.412451427	0.142768703
P2RX5	-0.367181424	0.196543996	-0.132583045	0.651389439
CD40LG	0.338758958	0.23610258	0.076371008	0.795256969
P2RX1	0.315960642	0.271117184	0.106592241	0.716841677
NFATC4	-0.295880736	0.304373217	-0.177865998	0.542956447
IKZF2	0.282264547	0.328199267	0.156645696	0.592795359
P2RX6	0.278211265	0.335488759	0.194192923	0.505898374
CXCL13	0.27322314	0.344582485	-0.147522977	0.614763318
CCL5	0.251364676	0.38601051	0.326356065	0.254788527
CTLA4	0.238696749	0.411171658	-0.309287178	0.281919505
KLRK1	0.23304681	0.422659331	0.318748739	0.266678134
P2RX4	0.229650921	0.429641614	-0.004047189	0.989044433
PRF1	0.222558935	0.444409022	0.17204863	0.556436998
TLR1	0.218078598	0.453866026	0.318243181	0.267479813
NFKB1	0.215864371	0.45857585	0.22799258	0.433072333
P2RY6	0.196756764	0.50018643	-0.01977631	0.94649979
P2RY14	0.195678645	0.502584729	0.064478038	0.826659341
ADORA1	0.182119792	0.533189646	0.135348831	0.644550551
CD69	0.173676857	0.55264968	0.253541798	0.38177042
TLR5	0.165255586	0.572355101	0.023184272	0.937295639
LAIR2	0.134640543	0.646299456	0.251081548	0.386563747
GZMA	0.112435417	0.701950259	0.116549968	0.691523248
EOMES	0.099828735	0.734195775	-0.009437107	0.974457248

IFNG	0.093966319	0.74933416	0.187135057	0.52177472
IL2	0.093891615	0.74952762	0.192188771	0.510384039
P2RY1	-0.072679761	0.804974816	-0.268723545	0.352901359
P2RX7	-0.051338345	0.861634043	-0.096817923	0.741959687
GZMB	-0.041526758	0.887908192	-0.011034731	0.970134699
GZMK	-0.007105822	0.980765936	0.062047384	0.833108563

Table S5: Classification performance of SVM-based interpretation of untargeted results in the exploratory study, related to Figure 4 and STAR Methods

		Training set	Test set
ESI+ mode (95% CI)	Mean specificity	1.0000 (1.0000-1.0000)	0.9862 (0.9852-0.9872)
	Mean sensitivity	1.0000 (1.0000-1.0000)	0.9526 (0.9514-0.9539)
	Mean accuracy	1.0000 (1.0000-1.0000)	0.9638 (0.9629-0.9647)
ESI- mode (95% CI)	Mean specificity	1.0000 (1.0000-1.0000)	0.9759 (0.9746-0.9771)
	Mean sensitivity	1.0000 (1.0000-1.0000)	0.8052 (0.8030-0.8073)
	Mean accuracy	1.0000 (1.0000-1.0000)	0.8621 (0.8606-0.8635)

Table S6: Information of the selected features in ESI+ and ESI- mode, related to Figure 4

Metabolite name	ion mode	Rank in SVM model	pKa (Strongest Acidic)	pKa (Strongest Basic)	Normal Concentrations in Blood (HMDB) [μm]
inosine	ESI+ / ESI-	5(+) / 1(-)	6.94	2.74	0.20 +/- 0.07
uridine	ESI-	3	9.7	-3	3.10 (2.90-3.30)
threonine	ESI-	4	2.21	9	127.7 +/- 41
phenylalanine	ESI-	6	2.47	9.45	78.1 +/- 20.5

The data source for pKa and normal blood concentration was the Human Metabolome Database (hmdb.ca).

Table S7: Initial and optimized conditions of the HILIC-MRM targeted quantitative assay, related to Figure 4 and Figure S8

**Initial conditions**		**Optimized conditions**	
Reconstitution Condition			
50% H_2O / 50% ACN		25% H_2O / 75% ACN	
Additives of Mobile Phase A			
5 mM ammonium acetate		1 mM ammonium acetate	
		0.1% formic acid	
Injection Volume (µl)			
5		2	
Dilution Factor			
1		4	
Source Temperature (°C)			
350		500	
Method Duration (min)			
23		19	
Gradient Program			
Time (min)	B.Conc (%)	Time (min)	B.Conc (%)
2	95	2	95
9	60	13	40
10	40	16	40
11	40	17.5	95
12	95	23	95
19	95		

Table S8: Detailed information of transitions in MRM assay for 4 selected features and their corresponding SIL-IS, related to Figure 4 and Figure S8

ID	Q1 Mass (Da)	Q3 Mass (Da)	Time (msec)	DP (volts)	CE (volts)
Inosine	269.1	137.0	10	40	18
Inosine	269.1	119.0	10	40	55
Uridine	245.1	113.0	10	80	12
Uridine	245.1	70.0	10	80	42
L-Phenylalanine	166.1	120.1	10	80	20
L-Phenylalanine	166.1	103.0	10	80	35
L-Threonine	120.0	74.0	10	60	15
L-Threonine	120.0	56.0	10	60	22
13C5-inosine	274.2	137.0	10	45	25
13C5-inosine	274.2	119.0	10	45	55
13C5-uridine	250.1	113.0	10	55	22
13C5-uridine	250.1	70.0	10	55	48
D8-L-Phenylalanine	174.1	128.1	10	50	22
D8-L-Phenylalanine	174.1	109.1	10	50	39
15N-L-Threonine	121.1	75.0	10	40	15
15N-L-Threonine	121.1	57.0	10	40	23

Table S9: Lower limits of detection (LLOD), lower limits of quantification (LLOQ), and linear ranges of four selected metabolites, related to Figure S10 and STAR Methods

Metabolite name	LLOD (ng/mL)	LLOQ (ng/mL)	Linear range (ng/mL)
Inosine	0.67	2.00	2-180
Uridine	26.67	80.00	80-7200
Phenylalanine	66.67	200.00	200-18000
Threonine	66.67	200.00	200-18000

Table S11: Performance of SVM-based modeling in the validation study, related to Figure 5

	Training cohort	Test cohort	Independent validation cohort
Accuracy	0.9409	0.9094	0.8929
Specificity	0.9071	0.9286	0.9286
Sensitivity	0.9576	0.8933	0.8714
AUC of ROC	0.9784	0.9502	0.9306
